# Tackling ALT-positive neuroblastoma: is it time to redefine risk classification systems? A systematic review with IPD meta-analysis

**DOI:** 10.1016/j.neo.2024.101106

**Published:** 2024-12-28

**Authors:** Marta Avinent-Pérez, Frank Westermann, Samuel Navarro, Amparo López-Carrasco, Rosa Noguera

**Affiliations:** a Department of Pathology, Medical School, University of Valencia, 46010 Valencia, Spain; b Neuroblastoma Genomics, German Cancer Research Center (DKFZ), Heidelberg, Germany; c Hopp Children's Cancer Center (KiTZ), Heidelberg, Germany; d Incliva biomedical health research institute, 46010 Valencia, Spain; e CIBER of Cancer (CIBERONC), 28029 Madrid, Spain

**Keywords:** Telomere maintenance mechanisms, Childhood cancer, Prognosis biomarker, Therapeutic strategies, Survival

## Abstract

**Background:**

The heterogeneous prognosis in neuroblastoma, shaped by telomere maintenance mechanisms (TMMs), notably the alternative lengthening of telomeres (ALT) pathway, necessitates a refined risk classification for high-risk patients. Current systems often lack precision, hindering tailored treatment approaches. This individual participant data (IPD) meta-analysis of survival among ALT-positive patients aims to improve risk classification systems, enhancing therapeutic strategies and patient outcomes.

**Methods:**

Following PRISMA-IPD guidelines, we conducted a comprehensive review of neuroblastoma patients retrieved from PubMed, Scopus, and Embase databases until March-2024. Patients were stratified into ALT-positive and TMM-negative subgroups. Overall and event-free survival probabilities were evaluated.

**Results:**

In our cohort of 293 patients (156 ALT-positive, 137 TMM-negative) obtained from eight different studies, ALT-positive individuals displayed lower survival rates than TMM-negative patients. Non-stage 4 ALT-positive patients had reduced overall and event-free survival probabilities compared to their TMM-negative counterparts, indicating potential misclassification. Stage 4 ALT-positive patients similarly showed poorer survival outcomes than non-stage 4 TMM-negative patients, underscoring the significance of ALT in patient prognosis.

**Conclusions:**

Our study highlights poorer outcomes in ALT-positive neuroblastoma patients, emphasizing the need to integrate TMM status into international risk classification guidelines. Standardizing TMM assessment is key for refining treatment strategies, considering the unique biology of ALT-positive patients.

## Background

Neuroblastoma, the most common extracranial solid tumor in childhood [[1], [2], [3]], exhibits significant clinical, biological, and histopathological heterogeneity [[4], [5], [6], [7]]. Clinically, low-risk patients often undergo spontaneous regression, while high-risk cases struggle, with survival rates hovering at approximately 50 % despite aggressive multimodal treatments [8,9].

Telomere maintenance mechanisms (TMMs) have emerged as critical determinants of prognosis in neuroblastoma patients [3,[8], [9], [10], [11], [12], [13], [14]], and are particularly implicated as pivotal factors contributing to therapy resistance among high-risk individuals [3,5,6]. TMMs operate through two main pathways: the telomerase-dependent pathway, driven by upregulation of telomerase reverse transcriptase (TERT), which typically occurs via MYCN amplification (MNA) or rearrangements at the TERT locus; and the often-overlooked telomerase-independent pathway, represented in approximately 15 % of cancers [11,15,16], and notably, in more than 20 % of neuroblastomas [11,17], known as alternative lengthening of telomeres (ALT) [4].

Accurate risk classification of patients into low, intermediate, or high-risk categories is essential for predicting prognosis and determining therapy strategies in neuroblastoma [4,14]. Although various international cooperative groups, including the International Society of Pediatric Oncology Europe Neuroblastoma Group (SIOPEN), the Children's Oncology Group (COG), and the German Society for Pediatric Oncology and Hematology (GPOH), employ slightly differing risk classification systems, they converge on assessing the following key factors to define high-risk neuroblastoma groups: the International Neuroblastoma Risk Group Staging System (INRGSS) [18] or the International Neuroblastoma Staging System (INSS) classification, age, and *MYCN* status [3,9,19].

Despite efforts to enhance risk classification systems, mortality rates among high-risk neuroblastoma patients remain alarmingly high [3,5,6]. To address this challenge, several research groups have strategically focused on categorizing high-risk neuroblastomas into three distinct molecular groups, all consistently associated with poor outcomes, based on TMM status (the MNA, ALT, and *TERT* rearrangements groups) [[4], [5], [6],^11^,^20^]. This targeted approach holds promise for developing more effective therapies tailored to the specific molecular characteristics of each subgroup [20].

The ALT pathway, which orchestrates telomere maintenance via intra- and inter-telomeric homologous recombination-dependent DNA replication, has been correlated with heightened replication stress and DNA damage at telomeres [15,20]. Mutations in the chromatin modifier *alpha thalassemia/mental retardation syndrome X-linked* (*ATRX*) gene, and more rarely in its binding partner *DAXX*, elevate replicative stress at telomeres and are predominantly correlated with ALT [21]. However, only about 55 % of ALT-positive neuroblastomas harbor *ATRX* alterations [3]. Also associated with ALT are mutations in p53 and RB1, which are crucial in the DNA damage response (DDR) [16]. Despite incomplete understanding of the molecular intricacies of ALT and the elusive triggers for its activation [8,15,21], several hallmarks of the ALT phenotype have been consistently observed. For instance, ALT-positive neuroblastomas are characterized by very long (up to >50kb) and heterogeneous telomeres [22,23], along with the presence of ALT-associated promyelocytic leukemia (PML) nuclear bodies (APBs) and abundant extrachromosomal telomeric repeat (ECTR) DNA that can include T-circles (double-stranded circular DNA) and C-circles (partially double-stranded circles in which the C-rich strand is intact), the levels of which correlate with ALT activity [16,24,25].

Evaluating ALT presents challenges since molecular markers linked to ALT may lack adequate sensitivity or specificity. For this reason, conducting a meta-analysis on ALT-positive neuroblastoma is essential to highlight the lack of consensus regarding biomarkers for its detection, as well as the limitations in the study designs that focus on it. While detecting ALT-associated mutations like *ATRX* alterations can contribute to determining ALT status, their sensitivity for ALT detection may prove insufficient, relegating their utility to supplementary roles alongside other diagnostic assays, such as C-circles [3,8]. However, it is noteworthy that a significant portion of studies rely on *ATRX* as the primary ALT marker [3,[26], [27], [28]], resulting in an underrepresentation of ALT-positive patients in the existing research [3]. Another issue is that many of the published studies on TMMs tend to combine telomerase-positive and ALT-positive patients, often highlighting the worse short-term survival of telomerase-positive patients [14]. In addition, few studies offer individualized clinic-biological information on neuroblastoma patients classified by their specific telomere elongation mechanism (individual participant data, IPD). This meta-analysis consolidates valuable IPD on ALT-positive and TMM-negative neuroblastoma patients from eight publications, underscoring the importance of making this information accessible to the scientific community.

Through this meta-analysis, our objective is to elucidate the true significance of ALT activation in the prognosis of neuroblastoma patients. By evaluating its impact on both event-free and overall survival, we aim to underscore its importance in refining risk stratification protocols, redefining high-risk neuroblastoma groups, and ultimately improving patient outcomes.

## Methods

This systematic review followed the PRISMA-IPD statement [29], based on a predefined protocol (Supplementary data Figures S1 and S2, Tables S1 and S2).

### Sub-cohorts and selection criteria

Patients of any age diagnosed with neuroblastoma or nodular ganglioneuroblastoma from observational studies published in English were included in our meta-analysis and formed the Integrated Participant Cohort (IPC). They were divided into two sub-cohorts: ALT-positive and TMM-negative (Table 1).

**Table 1:** Studies included in the meta-analisis, number of cases obtained from each study according to their telomere status, and technics applied to analyze telomeres length or content and classify cases as ALT+ or TMM-.

Study N	Study or Subgroup	N ALT+	N TMM-	Telomeres analysis	ALT+ assessment	TMM- confirmation
1	Koneru B., et al. Cancer Res. 2020;80(12):2663 [11]	36	34	WGS, TRF	ATRX: WGS. C-circles	TERT exp, TRAP
2	Kurihara S., et al. J Pediatr Surg. 2014;49(12):1835 [28]	11	0	TRF	ATRX: NGS, SNPa	TRAP
3	Cheung N. K., et al. JAMA. 2012;307(10):1062 [26]	21	0	WGS, FISH	ATRX: WGS, qPCR, IHC	-
4	Hartlieb S.A., et al. Nat Commun. 2021;12(1):1269 [3]	60	28	WGS + TelomereHunter,	ATRX: WGS. C-circles, TERRA exp: RNA-seq + TelomereHunter	RNA-seq, TRAP, MS
5	Meeser A., et al., Cell Biosci. 2022;12(1):160 [8]	13	17	WGS + TelomereHunter, TVR singletons, TRF	ATRX: WGS. C-circles, APBs, TERRA exp: RNA-seq + TelomereHunter	RNA-seq, TRAP
6	Lundberg G., et al. Genes Chr Cancer. 2011;50(4):250 [12]	10	0	FISH	ATRX: SNPa. APBs	-
7 and 8	Valentijn L. J., et al. Nat Genet. 2015;47(12):1411 [13]	6	58	WGS, TRF	ATRX: WGS, aCGH	TERT exp
	Van Gerven M. R., et al. Cancer Sci. 2022;113(6):2167 [27]					

N: number; ALT+: positive alternative lengthening of telomeres; TMM-: negative telomere maintenance mechanism WGS: whole genome sequencing; TRF: telomere restriction fragment; FISH: fluorescent in situ hybridization; TVR: telomere variant repeats; C-circles: partially double-stranded circles in which the C-rich strand is intact; aSNP: single nucleotide polymorphism array; NGS: next generation sequencing; qPCR: quantitative real-time polymerase chain reaction; IHC: immunohistochemistry; TERRA exp: telomeric long non-coding RNA expression; RNA-seq: RNA sequencing; APBs: ALT-associated promyelocytic leukemia nuclear bodies; aCGH: array-based comparative genomic hybridization; TRAP: telomeric repeat amplification protocol; MS: mass spectrometry.

**Fig. 1:**
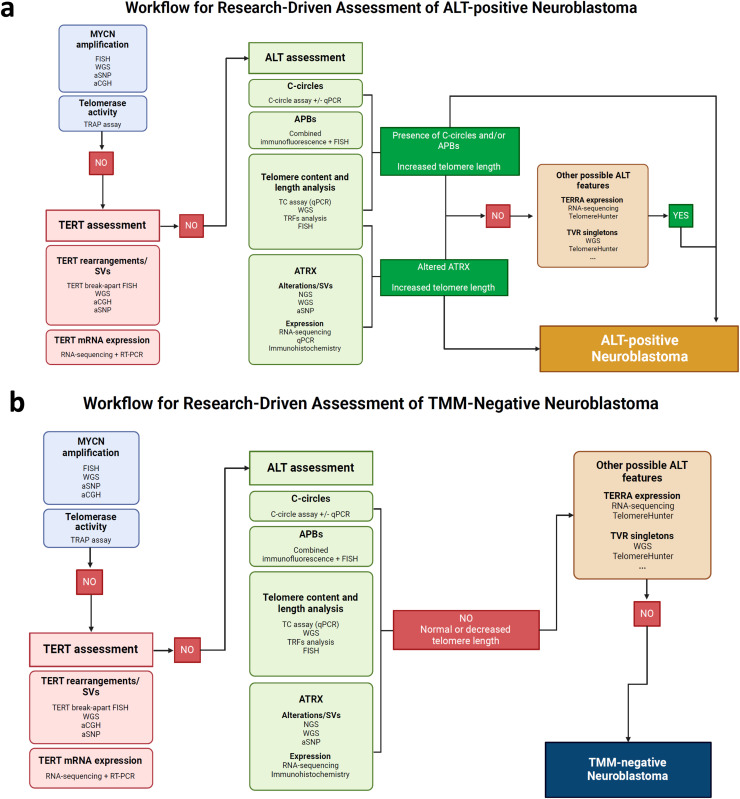
Definition of telomere status subgroups in this meta-analysis. Methods applied to classify ALT-positive (a) and TMM-negative (b) cases. C-circles: partially double-stranded circles in which the C-rich strand is intact; WGS: whole genome sequencing; aSNP: single nucleotide polymorphism array; aCGH: array-based comparative genomic hybridization; NGS: next generation sequencing; TRF: telomere restriction fragment; TRAP: telomeric repeat amplification protocol; qPCR: quantitative real-time polymerase chain reaction; APBs: ALT-associated promyelocytic leukemia nuclear bodies; FISH: fluorescent in situ hybridization; TVR: telomere variant repeats.

The ALT-positive sub-cohort (Fig. 1a) comprised cases from studies in which ALT presence was assessed or confirmed using at least one ALT biomarker (C-circles and/or APBs). In studies that used ATRX as the primary biomarker, only cases with both ATRX alterations and increased telomere length were included. In our cohort, telomeres were classified as long if Terminal Restriction Fragments (TRF) were greater than 15 kb, or if whole-genome sequencing (WGS) showed a difference in telomere reads between the tumor and matched germline exceeding 0.05. A low *TERT* expression or low telomerase activity was also determined to exclude TMM-positive cases via conventional telomerase activation in all the studies, except for study 3 [26] and 6 [12], which confirmed ALT by *ATRX* low expression and/or absence of MNA and *TERT* aberrations. Median TERT mRNA expression was the cutoff to define TERT high and low groups [11]. In the study 5 [8], a threshold of log2 expression values at 7.58 was defined as the lowest expression value having a probability ≥95 % to fall within the tumors with TERT/MYCN alteration [8]. Telomerase activity was considered negative or low if TRAP absorbance was lower than 0.2 [11] or, if expressed as Total Product Generated (TPG) lower than 10 [28]. In our meta-analysis, approximately 71 % of patient tumors underwent C-circle assays for ALT screening (Supplementary data; Figure S3).

In the TMM-negative sub-cohort (Fig. 1b), the inclusion criteria was a lack of MNA, of *TERT* rearrangements or structural variations (SVs) and of ATRX mutations or SVs, together with short telomeres and low TERT expression and/or low telomerase activation. No TMM-negative cases were selected from the two studies (3 [26] and 6 [12]) for which TERT expression and telomerase activity were not assessed. Patients with heterogeneous tumors (MNA/ALT or *TERT*/ALT) were omitted as part of the exclusion criteria.

Regarding outcomes, studies reporting survival outcomes and/or individual participant data (IPD) on survival were included regardless of whether survival data was explicitly analyzed by the authors. Studies with a median follow-up time of more than 5 years were specifically chosen due to the protracted clinical course typically experienced by ALT-positive neuroblastoma patients, with events usually occurring over 5 years from diagnosis [3].

Given that the ALT-positive subgroup predominantly consisted of patients categorized as stage 4 according to INSS criteria (n=131), while only 35.5 % (n=49) of TMM-negative patients fell into this category, we opted to conduct survival analysis emphasizing risk classification within both subgroups. This decision aimed to mitigate potential biases in our analysis associated with the inherent clinical and biological characteristics of the patients in each sub-cohort. Indeed, three studies (Supplementary data on individual studies; studies 1 [11], 3 [26] and 6 [12]) exclusively targeted high-risk neuroblastoma patients, further justifying our approach.

Based on the INSS reported for each patient, two additional sub-cohorts were therefore formed: stage 4 and non-stage 4. These sub-cohorts were further stratified into ALT-positive and TMM-negative for comparative analysis between the subgroups. The non-stage 4 sub-cohort included patients classified as stage 1, 2, 3, and 4S, the last group included as non-stage 4 since 4S-neuroblastoma patients under one year old are known to be a distinct subgroup linked to spontaneous regression in infants [3]. Importantly, none of the 4S patients in our cohort were positive for ALT.

### Outcomes

Comparisons of outcomes were made across the following subgroups: ALT-positive versus TMM-negative sub-cohorts, stage 4 ALT-positive versus stage 4 TMM-negative sub-cohorts, and non-stage 4 ALT-positive versus non-stage 4 TMM-negative sub-cohorts.

The primary outcome encompassed 5- and 10-year overall survival probabilities during follow-up. Overall survival was defined consistently across studies as the duration from initial diagnosis until the time of death from any cause, with alive patients censored at their last contact. Median overall survival time also was analyzed (Supplementary data; Tables S3a, S4a).

The secondary outcome consisted of 5- and 10-year event-free survival probabilities during follow-up, or 7.5-year probabilities if longer follow-up data were unavailable. Event-free survival was defined across studies as the duration from initial diagnosis to either the first occurrence of relapse, progressive disease, secondary malignancy, and/or death. Median event-free survival time was also analyzed (Supplementary data; Tables S3b, S4b). It should be noted that for the event-free survival rate analysis, the ALT-positive sub-cohort included 145 patients, while the TMM-negative sub-cohort comprised 79 patients due to incomplete data on event-free survival status across certain studies (Supplementary data on individual studies).

### Search strategy and study selection

The PubMed, Scopus and Embase databases were systematically searched up to March 22nd, 2024. The search strategy involved various combinations of terms, including: (“Telomere Maintenance Mechanisms” OR “Alternative Lengthening of Telomeres” OR “Telomere”) AND (“Neuroblastoma”) AND (“overall survival” OR “survival” OR “mortality” OR “event free survival” OR “prognosis” OR “risk classification” OR “clinical outcome”). Although this strategy may appear broad, it was formulated to address the scarcity of research on ALT in neuroblastoma and its impact on patient outcomes. Consequently, it allowed us to identify studies that not only indirectly provided data on ALT and patient outcomes, but also analyzed crucial factors for defining the TMM-negative sub-cohort: *MYCN* and *TERT* status.

A total of 358 studies were identified through database searching following our strategy. After 28 duplicates had been removed, 313 studies were excluded (mainly based on title and abstract) for not meeting the criteria of observational study and/or ALT status analysis. A total of 17 articles finally underwent eligibility assessment based on our inclusion criteria, but some did not provide adequate IPD. Among these, IPD were obtained from a total of eight studies (Table 1) for the first outcome (with study 8 [27] providing updated data from the ALT-positive patients included in the study 7 [13]), and from six studies for the second outcome (study 2 [28] did not provide data about event-free survival).

### Data collection and extraction

Most included studies were from 2020–2022 and had long follow-up times over 5 years; therefore, we primarily sourced IPD from their supplementary materials.

For Study 2 [28], conducted in 2014, we attempted to procure updated IPD via email. Unfortunately, no response was forthcoming. However, considering its extended follow-up period and alignment with our inclusion criteria, we incorporated patients from this study into the ALT-positive sub-cohort. For Study 7 [13], conducted in 2015, we included its IPD as the study's extensive follow-up period met our criteria. Moreover, in 2022 van Gerver et al. [27] presented updated survival data for the ALT-positive patients in Study 7, so we incorporated their latest findings. For Study 6 [12], conducted in 2011, we obtained the IPD from the included patients through the NeuPAT database [30].

Following our selection criteria for TMM-negative patients, no patients from studies 3 [26] or 6 were included in the TMM-negative sub-cohort, as *TERT* status was not provided.

The following data were extracted and imported into an Excel spreadsheet for all patients: ALT status, ALT biomarker, age, sex, INSS stage, tumor type, status (overall survival and event-free survival), and follow-up time. Additionally, the following data were collected when available: risk classification, ploidy, and for ALT-positive patients ATRX status. A thorough validation of IPD was conducted to ensure reliability in survival analysis across the studies. This involved verifying sequence generation to ensure logical event progression. Additionally, data consistency and completeness were meticulously checked to prevent missing or incomplete survival data. This validation process ensured the integrity of the data for robust survival analysis in the meta-analysis.

### Study quality assessment

The quality of each individual study (n=8) was evaluated using the Newcastle-Ottawa Scale (NOS) for cohort studies, analyzing accordingly the domains of study group selection and comparability, and outcome. Subsequently, the results were converted to AHQR standards to categorize them as good, fair, or poor quality. All our studies were found to meet the criteria for good quality, with scores ranging from 8 to 9 points.

In addition to evaluating study quality, heterogeneity among the included studies was visually assessed using stacked bar plots (Supplementary data; Fig. S4) as part of our methodological approach.

### Statistical analysis

The association of ALT presence with clinical features such as age, INSS stage, sex, risk classification, and ploidy were assessed using Chi-squared tests and Fisher's exact tests in R (version 4.3.3).

Survival curves were constructed using Kaplan-Meier analysis with the survminer package (version 0.4.9) and visualized using the ggsurvfit package (version 1.0.0). P values for survival analysis were determined using log-rank tests.

X-year survival analysis and median survival time with their corresponding 95 % confidence intervals (CI) were computed using the same version of R and directly obtained from the survfit object. Tables summarizing the results were generated using the gtsummary package (version 1.7.2).

Cox regression analysis was employed to assess overall survival- and event-free survival-related predictors (Wald forward and backward stepwise methods), hazard ratios (HR), p values and CI were determined using SPSS (version 28.0.1.1).

## Results

### Characteristics of Integrated Participant Cohort (IPC) patients

The IPC included a total of 293 patients divided into two distinct sub-cohorts. The ALT-positive sub-cohort constituted 53.2 % of patients (n=156), while the TMM-negative sub-cohort accounted for 46.8 % (n=137) of total patients (Table 2). Concerning age, 96.2 % of patients in the ALT-positive sub-cohort were diagnosed at 18 months or older, contrasting with 62 % of patients in the TMM-negative sub-cohort who were younger than 18 months at diagnosis. Both sexes were equally represented in the two sub-cohorts. Among the 293 patients, 277 were diagnosed with neuroblastoma, with only 12 diagnosed with nodular ganglioneuroblastoma, the majority being ALT-positive patients (n=9).

**Table 2:** Clinical-biological characteristics of patients in the IPC.

		Total cohort		ALT positive		TMM negative		p-value^f^
		n^e^	%^e^	n^e^	%^e^	n^e^	%^e^	
Total		293	100	156	53.2	137	46.8	
Age^b^	< 18 months	91	28.7	6	3.8	85	62	<0.001
	≥ 18 months	202	71.3	150	96.2	52	38	
Tumor type	NB	277	94.7	144	92.3	133	97.1	
	GNB, nodular	12	4	9	5.8	3	2.2	
	NA	4	1.3	3	1.9	1	0.7	
INSS	Stage 4	179	61.1	130	83.3	49	35.8	<0.001
	Non-Stage 4^c^	114	38.9	26	16.7	88	64.2	
Sex	Female	124	42.2	65	41.7	59	43	0.902
	Male	169	57.8	91	58.3	78	57	
Risk classification (n=168)^d^	HR	104	65.4	85	80.2	19	30.6	<0.001
	IR	26	14	12	11.3	14	22.6	
	LR	37	20	8	7.5	29	46.8	
	NA	1	0.6	1	0.9	0	0	
Ploidy (n=158)	Hyperdiploid	76	48.1	52	54.2	24	38.7	0.141
	Diploid	77	48.7	41	42.7	36	58.1	
	Complex	5	3.2	3	3.1	2	3.2	
ATRX status (n=134)	Altered	97	30.3	97	62.2	0	0	
	Wildtype	37	23.7	37	23.7	0	0	

^a^ n refers to the number of patients.

b Age at diagnosis.

c Non-stage 4 includes: stage 1 (IPCn = 29, ALTposn = 9, TMMnegn = 20), stage 2 (IPCn = 35, ALTposn = 9, TMMnegn = 26), stage 3 (IPCn = 16, ALTposn = 8, TMMnegn = 8) and stage 4S (IPCn = 34, ALTposn = 0, TMMnegn = 34).

d Risk classification abbreviations: HR = high-risk, IR = intermediate-risk, LR = low-risk, and NA = not available.

e Values are depicted both in terms of absolute patient counts (n) and as proportions ( %) relative to the total number of patients within each group (total cohort, ALT positive, TMM negative).

f P-values were calculated using Chi-squared and Fisher's exact tests.

Remarkably, 83.3 % of ALT-positive patients were diagnosed at stage 4 according to INSS criteria, whereas most of the TMM-negative sub-cohort (64.2 %) were classified as non-stage 4 at diagnosis. It is worth highlighting again that three of the studies only included high-risk patients, potentially leading to an under-representation of non-stage 4 ALT-positive patients in our cohort.

**Fig. 2:**
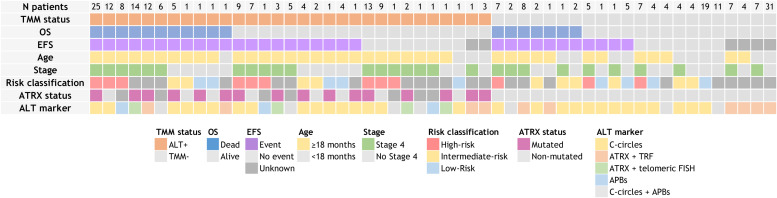
Telomere maintenance mechanisms of the neuroblastoma cohort and clinical-molecular landscape overview. The top panels show the number (N) of patients with the same clinical-molecular features, followed by telomere maintenance mechanisms (TMM) status. The central panels show clinical characteristics including overall survival (OS), event free survival (EFS), age, stage and risk classification. ATRX status, and main ALT marker applied to study telomere status are shown in the bottom panels. See figure legend for color codes. ALT+: positive alternative lengthening of telomeres; TMM-: negative TMM; C-circles: partially double-stranded circles in which the C-rich strand is intact; TRF: telomere restriction fragment; FISH: fluorescence in situ hybridization; APBs: ALT-associated promyelocytic leukemia nuclear bodies.

Risk classification was provided for 168 patients across various studies employing different risk classification systems. Similar results to the stage 4 findings were nonetheless observed, with 80.2 % of ALT-positive patients designated as high-risk, and 69.4 % of the TMM-negative sub-cohort classified as non-high risk (Fig. 2).

In our cohort, 62,2 % of ALT-positive patients had ATRX alterations. This statistic may not be fully representative of the ALT-positive population, however, as three studies (studies 2 [28], 3 [26] and 7 [13], Supplementary data on individual studies;) solely defined ALT-positive patients based on ATRX status and telomere length or content.

Further details regarding the individual studies, including detailed patient characteristics and specific analyses conducted within each study, are available in the Supplementary data (Supplementary data on individual studies; Figures S5–S11, Table S5–S11).

### Overall survival probability in ALT-positive vs. TMM-negative patients

Kaplan-Meier analysis in overall survival revealed significantly distinct survival outcomes between the ALT-positive and TMM-negative sub-cohorts (p < 0.001). The 5-year and 10-year overall survival probabilities varied across subgroups, with the ALT-positive sub-cohort showing a significantly lower 5-year overall survival probability of 53 % compared to 77 % in the TMM-negative sub-cohort.

**Fig. 3:**
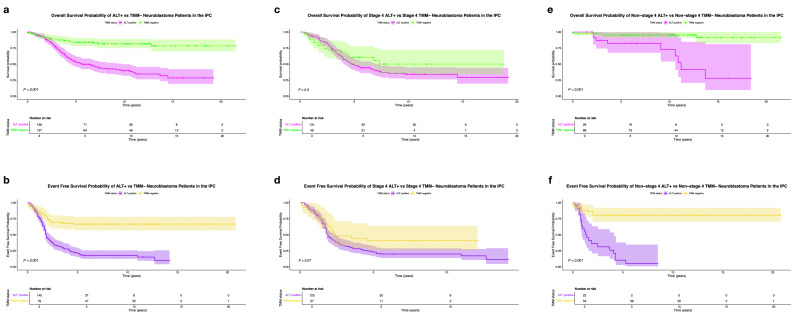
Survival probability between ALT-positive and TMM-negative patients in the IPC: (a) Overall survival, OS, (b) Event-free survival, EFS; survival probability between stage 4 ALT-positive and TMM-negative patients in the IPC: (c) Overall survival, OS, (d) Event-free survival, EFS; survival probability between non-stage 4 ALT-positive and TMM-negative patients in the IPC: (e) Overall survival, OS, (f) Event-free survival, EFS.

Pivotally, the overall survival probability of the ALT-positive sub-cohort decreased to 40 % at 10 years, while in the TMM-negative sub-cohort it remained above 70 % (Fig. 3a and Supplementary data, Table S3a).

Median survival was 5.6 years in the ALT-positive sub-cohort (Supplementary data; Table S4a). The nature of the TMM-negative sub-cohort precluded calculation of median survival time, as fewer than 50 % of patients experienced the event of interest within the observed timeframe. Therefore, median survival time estimation was not applicable for this subgroup.

As can be observed in Supplementary Data, Figure S12a, overall survival probability of ALT-positive and TMM-negative patients was compared with TERT-positive, and heterogeneous TERT/ALT sub-cohorts (see individual patients characteristics of the sub-cohorts in the Supplementary Excel). By 10-years the overall survival probabilities for both the ALT-positive and TERT-positive subgroups were similar (40 %).

### Event-free survival probability in ALT-positive vs. TMM-negative patients

For event-free survival probability analysis, the ALT-positive subgroup comprised 145 patients, while the TMM-negative subgroup consisted of 79 patients, due to incomplete data on event-free survival status across certain studies (studies 2 [28] and 7 [13]).

ALT-positive patients had a significantly shorter event-free survival than TMM-negative patients. The 5-year event-free survival probability in the ALT-positive subgroup was 21 % compared to 67 % in the TMM-negative subgroup (Fig. 3b and Supplementary data, Table S3b). Importantly, while the event-free survival probability of the TMM-negative subgroup remained the same at 10 years, this probability decreased to 18 % in the ALT-positive subgroup, with a median event-free survival time of 1.8 years (Supplementary data; Table S4b).

The comparative analysis with the TERT-positive and the heterogeneous TERT/ALT subgroups (Figure S12b) revealed that, by 10-years, the event-free survival probability was notably lower in the ALT-positive sub-cohort (18 %) than in the TERT-positive one (29 %).

### Overall survival probability in stage 4 ALT-positive patients vs. stage 4 TMM-negative patients

As most patients in the ALT-positive sub-cohort were classified as stage 4 according to the INSS criteria (n=131), whereas only 35.5 % of patients in the TMM-negative subgroup were categorized as higher risk (n=49), we opted to conduct survival analysis focusing on risk classification from the patients in both sub-cohorts. This decision aimed to mitigate potential biases associated with the intrinsic tumor characteristics of the respective neuroblastoma subgroups in the preceding analysis.

Stage 4 ALT-positive patients exhibited a lower 5-year overall survival rate of 48 % than stage 4 TMM-negative patients, who demonstrated a 5-year overall survival probability of 61 % (Fig. 3c and Supplementary data, Table S3a). Over a longer period, the 10-year survival probability of the stage 4 ALT-positive subgroup decreased to 34 %, while in the TMM-negative subgroup it also declined to 50 %.

Median survival times also exhibited a between-subgroup difference, with 4.8 years observed in the ALT-positive subgroup compared to 7.3 years in the TMM-negative subgroup (Supplementary data; Table. S4a).

### Event-free survival probability in stage 4 ALT-positive patients vs. stage 4 TMM-negative patients

The stage 4 ALT-positive subgroup in our cohort for analyzing event-free survival comprised 123 patients, while the TMM-negative group included data drawn from 27 stage 4 patients. Nonetheless, a difference among the subgroups could be found. In stage 4 patients with ALT positivity, 5-year event-free survival stood at 23 %. Contrarily, those without TMM demonstrated a notably higher rate, with 41 % 5-year event-free survival levels maintained consistently over time (Fig. 3d and Supplementary data, Table S3b). However, for the ALT-positive subgroup, this probability declined further, reaching 20 % by the end of the 10-year period. Similarly, the median event-free survival time for the ALT-positive subgroup was 1.9 years, in contrast to 2.4 years for the TMM-negative subgroup (Supplementary data; Table S4b).

### Overall survival probability in non-stage 4 ALT-positive patients vs. non-stage 4 TMM-negative patients

Non-stage 4 ALT-positive patients also exhibited a significantly lower overall survival probability than those without TMM alterations (p < 0.001). Within our cohort, 26 ALT-positive patients were categorized as non-stage 4. However, their 5-year overall survival probability stood at 82 %, declining to 73 % at 10 years, in contrast to non-stage 4 TMM-negative patients (n=88), in whom this probability remained stable at 95 % throughout the timeframe (Fig. 3e and Supplementary data, Table S3a). Median survival time could only be calculated for the ALT-positive subgroup, and was determined to be 11 years (Supplementary data; Table S4a).

### Event-free survival probability in non-stage 4 ALT-positive patients vs. non-stage 4 TMM-negative patients

An even more remarkable difference in event-free survival was found between non-stage 4 patient subsets (p < 0.001). Although the non-stage 4 ALT-positive sub-cohort for event-free survival analysis only accounted for 22 patients, the 5-year event-free survival probability was 10 %, compared to 81 % in the TMM-negative subgroup (Fig. 3f and Supplementary data, Table S3b). Significantly, the median event-free survival time was 1.4 years for this subgroup, while fewer than 50 % of patients in the TMM-negative subgroup experienced the event of interest within the observed timeframe (Supplementary data; Table S4b).

Cox regression proved that TMM status, in particular ALT activation, had a significant predictive value of event-free survival in non-stage 4 patients, independent of age at diagnosis, sex, tumor histopathology and ploidy, using both stepwise forward (HR = 5.52, p < 0.001, CI = 2.5-12.0) and backward Wald methods (HR = 2.86, p = 0.04, CI = 1.1-7.8).

## Discussion

Overall, the main finding of our IPD meta-analysis is the significantly inferior outcomes experienced by ALT-positive neuroblastoma patients compared to their TMM-negative counterparts, evident in both event-free and overall survival rates. Our study also reaffirms the notion that ALT-positive patients endure a protracted disease trajectory [14], marked by relapses persisting even a decade post initial diagnosis, which is reflected not only in the lower 10-year event-free survival probability of these sub-cohort compared to the TMM-negative one, but also to the TERT-positive patients.

A pivotal takeaway from our study is the urgent need to redefine neuroblastoma risk classification guidelines. Within our ALT-positive sub-cohort, a striking 16 % of patients were classified as non-stage 4 and consequently ruled out from high-risk status. However, these ostensibly "low risk" individuals displayed outcomes as dire as those labeled high-risk, a trend consistently observed across various studies [3,5]. This underscores the necessity of including ALT-positive patients in the high-risk category within risk classification protocols, thereby enabling more accurate treatment strategy decisions.

Additionally, we observed that stage 4 ALT-positive patients tended to experience worse outcomes than stage 4 TMM-negative patients. While not reaching statistical significance, this difference warrants further research with larger cohorts and more precise assessment of TMMs. Although several factors contribute to the heightened risk in stage 4 TMM-negative tumors, a noteworthy subset of high-risk TMM-negative neuroblastomas (constituting over 10 % of high-risk patients) display a distinct phenotype known as ever-shorter telomeres (EST) [11,25,31]. Despite lacking TMM activation, cell lines with the EST phenotype start with elongated telomeres and undergo progressive shortening [16,25], potentially contributing to sustained proliferation and poorer outcomes. This suggests that a certain proportion of stage 4 TMM-negative patients in our meta-analysis might harbor the EST phenotype, highlighting the need to consider telomere dynamics in neuroblastoma prognosis assessment and treatment strategies.

In recent years, significant effort has been dedicated to delineating the category of ultra-high-risk (UHR) neuroblastoma. As previously emphasized [9], the challenge lies in selecting the most appropriate metric for risk assessment, since definitions may vary among researchers, with criteria including refractory disease, death from disease within 18 months of diagnosis, or a 5-year event-free survival ranging between 10 % and 15 % [9]. Subgroups showing statistically significant differences in event-free survival can essentially be categorized as UHR or non-UHR [9]. In our study, we utilized time-to-event analysis to construct event-free and overall survival curves, revealing a notable disparity in both outcomes, particularly evident in event-free survival. Among ALT-positive neuroblastoma patients, 5-year event-free survival ranged from 10 % to 23 %, whereas in the TMM-negative sub-cohort event-free survival rates remained consistently above 41 %, peaking at 81 % across groups. This pronounced discrepancy suggests that ALT-positive neuroblastoma patients may warrant consideration as candidates for UHR classification. It is important to note that, as this study is a meta-analysis of substudies from various countries, it could be lacking an uniformly diagnosed and treated group of patients, being this a limitation when we estimated the probabilities of patient survival.

Furthermore, the presence of RAS/TP53 pathway alterations, particularly prevalent in ALT-positive relapsed neuroblastomas but consistently elevated across all ALT-positive neuroblastomas [3], has emerged as a critical determinant in characterizing UHR neuroblastoma among TMM-positive patients [8,10,17]. This emphasizes that defining ALT-positive neuroblastomas as more than just high-risk, but rather as UHR, is an idea worth considering.

As previously discussed, determining TMM status has become increasingly challenging and lacks standardization, posing a significant obstacle in accurately defining a TMM-negative cohort representative of this patient group. In our meta-analysis, meticulous selection criteria were devised to discern TMM-negative patients with maximal specificity. Nonetheless, a principal limitation must be acknowledged in terms of reliably identifying ALT-positive, and particularly TMM-negative patients. For instance, in most of the studies excluded from this meta-analysis, telomerase activation was defined solely by TERT overexpression resulting from MYCN amplification or TERT rearrangements. This approach may inadequately capture the telomerase-dependent subgroup, as TERT expression can occur via alternative pathways, potentially overlooking some TMM-positive cases [10,11]. Moreover, a subpopulation of MNA tumors has been reported to exhibit TERT expression levels as low as those found in ALT tumors [11]. Consequently, a few cases classified as TMM-positive and excluded from our study may, in fact, be false positives. Similarly, ALT pathway assessment varied greatly among studies, further complicating the establishment of a dependable cohort of ALT-positive patients. For example, APBs may not serve as optimal markers for ALT, as they are associated with long telomeres rather than being specific to the ALT mechanism, which can lead to false positives [11]. While C-circle detection is considered highly specific for ALT-positive cells, low signals may also be detected in normal tissues, particularly in blood samples [8,30]. Furthermore, relying solely on the C-circle assay for ALT detection may result in missed ALT-positive tumors [11]. Thus, both methods, used independently of each other, have limitations in reliably identifying ALT. Regarding TERT and ATRX expression, as well as telomere length or content, additional studies are needed to establish consistent thresholds across detection techniques [11,32].

It is also crucial to note that neuroblastomas often display intra-tumoral heterogeneity regarding TMMs, wherein certain cells exhibit telomerase activation while others showcase the characteristic ALT phenotype [16]. While we deliberately excluded cases with ambiguity or TERT/ALT heterogeneity from the ALT-positive subgroup to enhance clarity regarding the impact of ALT on neuroblastoma patient prognosis, we included these cases in an independent small sub-cohort to determine the influence of both ALT and TERT positivity on patients’ survival. The inherent variability within neuroblastomas underscores the need to standardize protocols for assessing TMM status. This standardization is pivotal for refining treatment decisions, especially in tackling the complexities presented by heterogeneous tumors. Furthermore, our study was limited by the scarcity of research examining the impact of ALT on survival. We advocate for future research efforts to include consensual guidelines, with well-established and comparable protocols, enabling better identification and inclusion of ALT-positive patients and accessible IPD for comprehensive analysis.

In a clinical context, we endorse recent recommendations for integrating TMM assessment into routine protocols, being the minimum information required to classify TMM in neuroblastoma the telomere length (TMM-positive versus TMM-negative), and the presence of APBs or C-circles to distinguish between ALT-positive cases from TMM-positive via telomerase (if possibly confirmed with TERT expression analysis). We recommend a practical approach based on FISH with different targets as due to its widespread adoption and rapid turnaround time: FISH analysis of *MYCN amplification, TERT rearrangements*, telomeres brightness and presence of APBs. For ambiguous cases requiring further clarification C-circles assays is proposed, with additional analyses, such as TERT and ATRX expression by qPCR or FISH (RNA in situ hybridization [32]), telomere length by southern blot and TERRA identification (specially applying RNA sequencing and TelomereHunter [3,8,17]). This approach, based on one technique with different targets, represents a pragmatic and economically viable solution for everyday clinical practice, especially when compared to whole-genome sequencing (plus TelomereHunter and telomere variant repeats singletons analysis) [3,8,17,33], Single Molecule Telomere Analysis and Telo-seq that use Oxford Nanopore Technologies [34,35], which although potentially valuable as future precise tests, are not yet universally accessible on an international scale and are encumbered by considerable cost implications [17].

In conclusion, we underscore the urgent necessity for international consensus on risk classification guidelines, with a specific focus on incorporating TMMs and particularly the ALT pathway as high-risk or UHR criteria, alongside standardized protocols for assessing TMM status. This harmonization is crucial for fostering robust research and facilitating clinical trials, particularly involving ALT-positive patients. In addition, the significance of developing tailored treatment strategies for ALT-positive neuroblastomas cannot be overstressed, given their unique attributes as slow-proliferating tumors inherently resistant to conventional high-risk neuroblastoma treatments. By implementing these initiatives, we anticipate a promising outlook for ALT-positive neuroblastoma patients.

## Funding information

This research was supported by a grant from the ISCIII and ERDF (PI20/01107) and CIBERONC (CB16/12/00484). FNB (Fundación Neuroblastoma) and Fundación CRIS (2023/188) fund AL-C contract. The founders were not involved in the research process nor in the preparation or submission of the article.

## CRediT authorship contribution statement

**Marta Avinent-Pérez:** Writing – original draft, Methodology, Investigation, Conceptualization. **Frank Westermann:** Writing – review & editing, Validation. **Samuel Navarro:** Writing – review & editing, Validation. **Amparo López-Carrasco:** Writing – original draft, Validation, Supervision, Investigation. **Rosa Noguera:** Writing – review & editing, Validation, Supervision, Conceptualization.

## Declaration of competing interest

The authors declare that they have no known competing financial interests or personal relationships that could have appeared to influence the work reported in this paper.

## Appendix Supplementary materials

Image, application 1

Image, application 2

Image, application 3

## References

[1] Brodeur G.M.Neuroblastoma (2003). Biological insights into a clinical enigma. Nat. Rev. Cancer.

[2] Maris J.M., Guo C., Blake D., White P.S., Hogarty M.D., Thompson P.M., Rajalingam V., Gerbing R., Stram D.O., Matthay K.K. (2001). Proceedings of the Medical and Pediatric Oncology.

[3] Hartlieb S.A., Sieverling L., Nadler-Holly M., Ziehm M., Toprak U.H., Herrmann C., Ishaque N., Okonechnikov K., Gartlgruber M., Park Y.G. (2021). Alternative lengthening of telomeres in childhood neuroblastoma from genome to proteome. Nat. Commun..

[4] Yu E.Y., Cheung N.K.V., Lue N.F. (2022). Connecting telomere maintenance and regulation to the developmental origin and differentiation states of neuroblastoma tumor cells. J. Hematol. Oncol..

[5] Ikegaki N., Shimada H. (2019). Subgrouping of unfavorable histology neuroblastomas with immunohistochemistry toward precision prognosis and therapy stratification. JCO Precis. Oncol..

[6] Shimada H., Ikegaki N. (2022). Genetic and Histopathological Heterogeneity of Neuroblastoma and Precision Therapeutic Approaches for Extremely Unfavorable Histology Subgroups. Biomolecules.

[7] Ponzoni M., Bachetti T., Corrias M.V., Brignole C., Pastorino F., Calarco E., Bensa V., Giusto E., Ceccherini I., Perri P. (2022). Recent advances in the developmental origin of neuroblastoma: an overview. J. Exp. Clin. Cancer Res..

[8] Meeser A., Bartenhagen C., Werr L., Hellmann A.M., Kahlert Y., Hemstedt N., Nürnberg P., Altmüller J., Ackermann S., Hero B.; (2022). Reliable assessment of telomere maintenance mechanisms in neuroblastoma. Cell Biosci.

[9] Morgenstern D.A., Bagatell R., Cohn S.L., Hogarty M.D., Maris J.M., Moreno L., Park J.R., Pearson A.D., Schleiermacher G., Valteau-Couanet D.; (2019). The challenge of defining “ultra-high-risk” neuroblastoma. Pediatr. Blood Cancer.

[10] Ackermann S., Cartolano M., Hero B., Welte A., Kahlert Y., Roderwieser A., Bartenhagen C., Walter E., Gecht J., Kerschke L. (2018). A mechanistic classification of clinical phenotypes in neuroblastoma. Science.

[11] Koneru, B.; Lopez, G.; Farooqi, A.; Conkrite, K.L.; Thinh, H.; Macha, S.J.; Modi, A.; Rokita, J.L.; Urias, E.; Davidson, H.; et al. Telomere maintenance mechanisms define clinical outcome in high-risk neuroblastoma. 2020, 80, 2663–2675, doi:10.1158/0008-5472.CAN-19-3068.Telomere. 10.1158/0008-5472.CAN-19-3068 PMC7313726 32291317

[12] Lundberg G, Sehic D, Länsberg JK, Øra I, Frigyesi A, Castel V, Navarro S, Piqueras M, Martinsson T, Noguera R G.D (2011). Alternative lengthening of telomeres—an enhancedchromosomal instability in aggressive non-MYCNamplified and telomere elongated neuroblastomas. Genes. Chromosomes Cancer.

[13] Valentijn L.J., Koster J., Zwijnenburg D.A., Hasselt N.E., Van Sluis P., Volckmann R., Van Noesel M.M., George R.E., Tytgat G.A.M., Molenaar J.J.; (2015). TERT rearrangements are frequent in neuroblastoma and identify aggressive tumors. Nat. Genet.

[14] Roderwieser A., Sand F., Walter E., Fischer J., Gecht J., Bartenhagen C., Ackermann S., Otte F., Welte A., Kahlert Y.; (2019). Telomerase is a prognostic marker of poor outcome and a therapeutic target in neuroblastoma. JCO Precis. Oncol.

[15] Cesare A.J., Reddel R.R (2010). Alternative lengthening of telomeres: models, mechanisms and implications. Nat. Rev. Genet.

[16] Sommer A., Royle N.J. (2020). ALT : a multi-faceted phenomenon. Genes (Basel).

[17] Werr L., Rosswog C., Bartenhagen C., George S.L., Fischer M. (2024). Telomere maintenance mechanisms in neuroblastoma: new insights and translational implications. *EJC Paediatr. Oncol*.

[18] Monclair T., Brodeur G.M., Ambros P.F., Brisse H.J., Cecchetto G., Holmes K., Kaneko M., London W.B., Matthay K.K., Nuchtern J.G.; (2009). The international neuroblastoma risk group (INRG) staging system: an INRG task force report. J. Clin. Oncol.

[19] Sokol E., Desai A.V. (2019). The evolution of risk classification for neuroblastoma. Children.

[20] George S.L., Parmar V., Lorenzi F., Marshall L.V., Jamin Y., Poon E., Angelini P., Chesler L. (2020). Novel therapeutic strategies targeting telomere maintenance mechanisms in high-risk neuroblastoma. J. Exp. Clin. Cancer Res..

[21] Zhang J.M., Zou L. (2020). Alternative lengthening of telomeres: from molecular mechanisms to therapeutic outlooks. Cell Biosci.

[22] Srinivas N., Rachakonda S., Kumar R. (2020). Telomeres and telomere length: a general overview. Cancers (Basel).

[23] Bryan T.M., Englezou A., Gupta J., Bacchetti S., Reddel R.R (1995). Telomere elongation in immortal human cells without detectable telomerase activity. EMBO J.

[24] Djos A., Thombare K., Vaid R., Gaarder J., Umapathy G., Reinsbach S.E., Georgantzi K., Stenman J., Carén H., Ek T.; (2023). Telomere maintenance mechanisms in a cohort of high-risk neuroblastoma tumors and its relation to genomic variants in the TERT and ATRX Genes. Cancers (Basel).

[25] Dagg R.A., Pickett H.A., Neumann A.A., Napier C.E., Henson J.D., Teber E.T., Arthur J.W., Reynolds C.P., Murray J., Haber M. (2017). Extensive proliferation of human cancer cells with ever-shorter telomeres. Cell Rep.

[26] Cheung N.V, Zhang J., Lu C., Bahrami A., Tickoo S.K., Heguy A., Pappo S., Federico S., Dalton J., Cheung I.Y.; (2012). Association of age at diagnosis and genetic mutations in patients with Neuroblastoma. JAMA.

[27] van Gerven M.R., Bozsaky E., Matser Y.A.H., Vosseberg J., Taschner-Mandl S., Koster J., Tytgat G.A.M., Molenaar J.J., van den Boogaard M. (2022). Mutational spectrum of ATRX aberrations in neuroblastoma and associated patient and tumor characteristics. Cancer Sci.

[28] Kurihara S., Hiyama E., Onitake Y., Yamaoka E., Hiyama K (2014). Clinical features of ATRX or DAXX mutated neuroblastoma. J. Pediatr. Surg.

[29] Stewart L.A., Clarke M., Rovers M., Riley R.D., Simmonds M., Stewart G., Tierney J.F. (2015). Preferred reporting items for a systematic review and meta-analysis of individual participant data: the PRISMA-IPD statement. JAMA - J. Am. Med. Assoc..

[30] Villamón E., Piqueras M., Meseguer J., Blanquer I., Berbegall A.P., Tadeo I., Hernández V., Navarro S., Noguera R. (2013). NeuPAT: An intranet database supporting translational research in neuroblastic tumors. Comput. Biol. Med..

[31] Stainczyk S.A., Westermann F. (2022). Neuroblastoma—Telomere maintenance, deregulated signaling transduction and beyond. Int. J. Cancer.

[32] Zhao M., Guan Z., Gong L., Liu F., Gu W., Liu L., Jiang K., Cai J., Feng C., Kuick C.H.; (2023). Rapid detection of telomerase expression of neuroblastoma in paraffin-embedded tissue: combination of in situ hybridisation and quantitative PCR. Pathology.

[33] Muyas F., Rodriguez M.J.G., Cascão R., Afonso A., Sauer C.M., Faria C.C., Cortés-Ciriano I., Flores I. (2024). The ALT pathway generates telomere fusions that can be detected in the blood of cancer patients. Nat. Commun.

[34] Raseley K., Jinwala Z., Zhang D., Xiao M. (2023). Single-molecule telomere assay via optical mapping (SMTA-OM) can potentially define the ALT positivity of cancer. Genes (Basel).

[35] Schmidt T.T., Tyer C., Rughani P., Haggblom C., Jones J.R., Dai X., Frazer K.A., Gage F.H., Juul S., Hickey S.; (2024). High resolution long-read telomere sequencing reveals dynamic mechanisms in aging and cancer. Nat. Commun.

