# Supplementary material for: Tackling ALT-positive neuroblastoma: is it time to redefine risk classification systems? A systematic review with IPD meta-analysis
Source: Neoplasia. 2024 Dec 28;60:101106. doi: 10.1016/j.neo.2024.101106 (PMC11743311; doi:10.1016/j.neo.2024.101106)
Supplement: Supplementary file 2 [file mmc2.pdf]

Supplementary data

Tackling ALT-positive neuroblastoma: is it time to redefine risk classification systems? A systematic review with IPD meta-analysis

Marta Avinent-Pérez[1,2], Frank Westermann[2,3], Samuel Navarro[1, 4, 5], Amparo López-Carrasco[1, 4, 5, ✉], and Rosa Noguera[1, 4, 5, ✉]

Author Affiliations

[1]Department of Pathology, Medical School, University of Valencia, 46010, Valencia, Spain. [2]Neuroblastoma Genomics, German Cancer Research Center (DKFZ), Heidelberg, Germany. [3]Hopp Children's Cancer Center (KiTZ), Heidelberg, Germany. [4]Incliva biomedical health research institute, 46010, Valencia, Spain [5]CIBER of Cancer (CIBERONC), 28029, Madrid, Spain.

Corresponding authors emails:

✉ malopez@incliva.es, ORCID: 0000-0001-9202-2597

✉ rosa.noguera@uv.es, ORCID: 0000-0003-4546-7459

Table of contents

Supplementary Figures

Fig. S1. Prisma-IPD flow diagram from main outcome 1: overall survival

Fig. S2. Prisma-IPD flow diagram from main outcome 2: event-free survival

Fig. S3. Principal biomarkers for ALT assessment in the Integrated Participant Cohort (IPC).

Fig. S4. Stacked bar plot assessing heterogeneity across studies (S1–S7)

Supplementary Tables

Table S1. Prisma-IPD checklist.

Table S2. Table lists patient ID, data availability, ALT status by biomarker, age, sex, INSS stage, risk classification, tumor type, ploidy, event-free and overall survival status, and follow-up times for patients forming both ALT-positive and TMM-negative sub-cohorts.

Table S3. 5-year and 10-year overall and event-free survival probabilities across studies and in the IPC.

Table S4. Median overall and event-free survival across studies and in the IPC.

Supplementary data on individual studies

Study 1: Koneru B, Lopez G, Farooqi A, Conkrite KL, Nguyen TH, Macha SJ, et al. Telomere maintenance mechanisms define clinical outcome in high-risk neuroblastoma. Cancer Res. 2020;80(12):2663–75.

Table S5. Clinical-biological characteristics of S1 patients

Fig. S5. Overall and event-free survival curves of S1

Study 2: Kurihara S, Hiyama E, Onitake Y, Yamaoka E, Hiyama K. Clinical features of ATRX or DAXX mutated neuroblastoma. J Pediatr Surg [Internet]. 2014;49(12):1835–8.

Table S6. Clinical-biological characteristics of S2 patients

Fig. S6. Overall survival curve of S2

Study 3: Cheung N-KV. Association of age at diagnosis and genetic mutations in patients with neuroblastoma. JAMA. 2012;307(10):1062.

Table S7. Clinical-biological characteristics of S3 patients

Fig. S7. Overall and event-free survival curves of S3

Study 4: Hartlieb SA, Sieverling L, Nadler-Holly M, Ziehm M, Toprak UH, Herrmann C, et al. Alternative lengthening of telomeres in childhood neuroblastoma from genome to proteome. Nat Commun. 2021;12(1).

Table S8. Clinical-biological characteristics of S4 patients

Fig. S8. Overall and event-free survival curves of S4 patients

Study 5: Meeser A, Bartenhagen C, Werr L, Hellmann A-M, Kahlert Y, Hemstedt N, et al. Reliable assessment of telomere maintenance mechanisms in neuroblastoma. Cell Biosci. 2022;12(1).

Table S9. Clinical-biological characteristics of S5 patients

Fig. S9. Overall and event-free survival curves of S5

Study 6: Lundberg G, Sehic D, Länsberg J-K, Øra I, Frigyesi A, Castel V, et al. Alternative lengthening of telomeres—An enhanced chromosomal instability in aggressive non-*MYCN* amplified and telomere elongated neuroblastomas. Genes Chromosomes Cancer. 2011;50(4):250–62.

Table S10. Clinical-biological characteristics of S6 patients

Fig. S10. Overall and event-free survival curves of S6

Study 7: Valentijn LJ, Koster J, Zwijnenburg DA, Hasselt NE, van Sluis P, Volckmann R, et al. TERT rearrangements are frequent in neuroblastoma and identify aggressive tumors. Nat Genet. 2015;47(12):1411–4.

Survival data from ALT-positive patients were updated in Study 8: van Gerven MR, Bozsaky E, Matser YAH, Vosseberg J, Taschner-Mandl S, Koster J, et al. Mutational spectrum of *ATRX* aberrations in neuroblastoma and associated patient and tumor characteristics. Cancer Sci. 2022;113(6):2167–78.

To enhance clarity and facilitate data analysis, both studies were combined into Study 7 in the supplementary data (e.g., Fig. S4).

Table S11. Clinical-biological characteristics of S7 patients (TMM-negative subcohort) and S8 patients (ALT-positive subcohort)

Fig. S11. Overall and event-free survival curves of S7

Fig. S12. Survival probabilities among ALT-positive, TMM-negative, TERT-positive and TERT/ ALT heterogeneous patients.
Overall and event-free survival probabilities of ALT-positive and TMM-negative groups were compared with those of 63 TERT-positive patients from studies 1 [11], 4 [3], 5 [8] and 7 [13], and of 5 heterogeneous tumors TERT/ALT from study 5 [8]. Heterogeneous tumors MYCN/TERT and MYCN/ALT were excluded from the analysis due to the small number of cases (3) and the lack of survival data from the substudies, respectively. Specifically, the 63 TERT-positive patients were included in the study to calculate 5- and 10-year overall survival probability, and 50 of them to calculate the event-free survival probability. From the 5 heterogeneous tumors TERT/ALT, overall survival probability was obtained at 3-years and 5-years, and the event-free survival probability at 1-year and 3-years after.

Supplementary figures

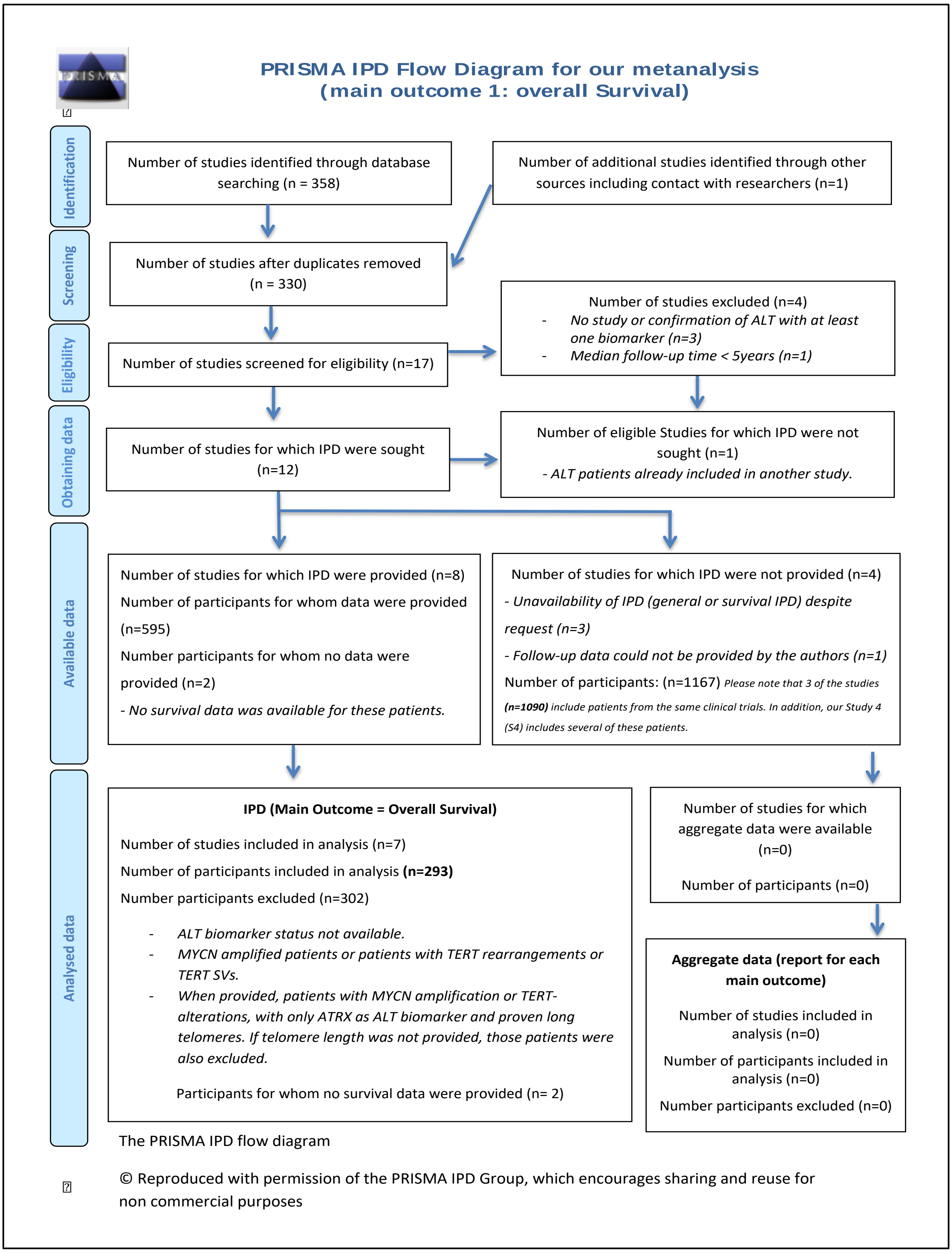

Fig. S1. Prisma-IPD flow diagram from main outcome 1: overall survival. A total of seven studies and 293 patients were included in the IPD meta-analysis.

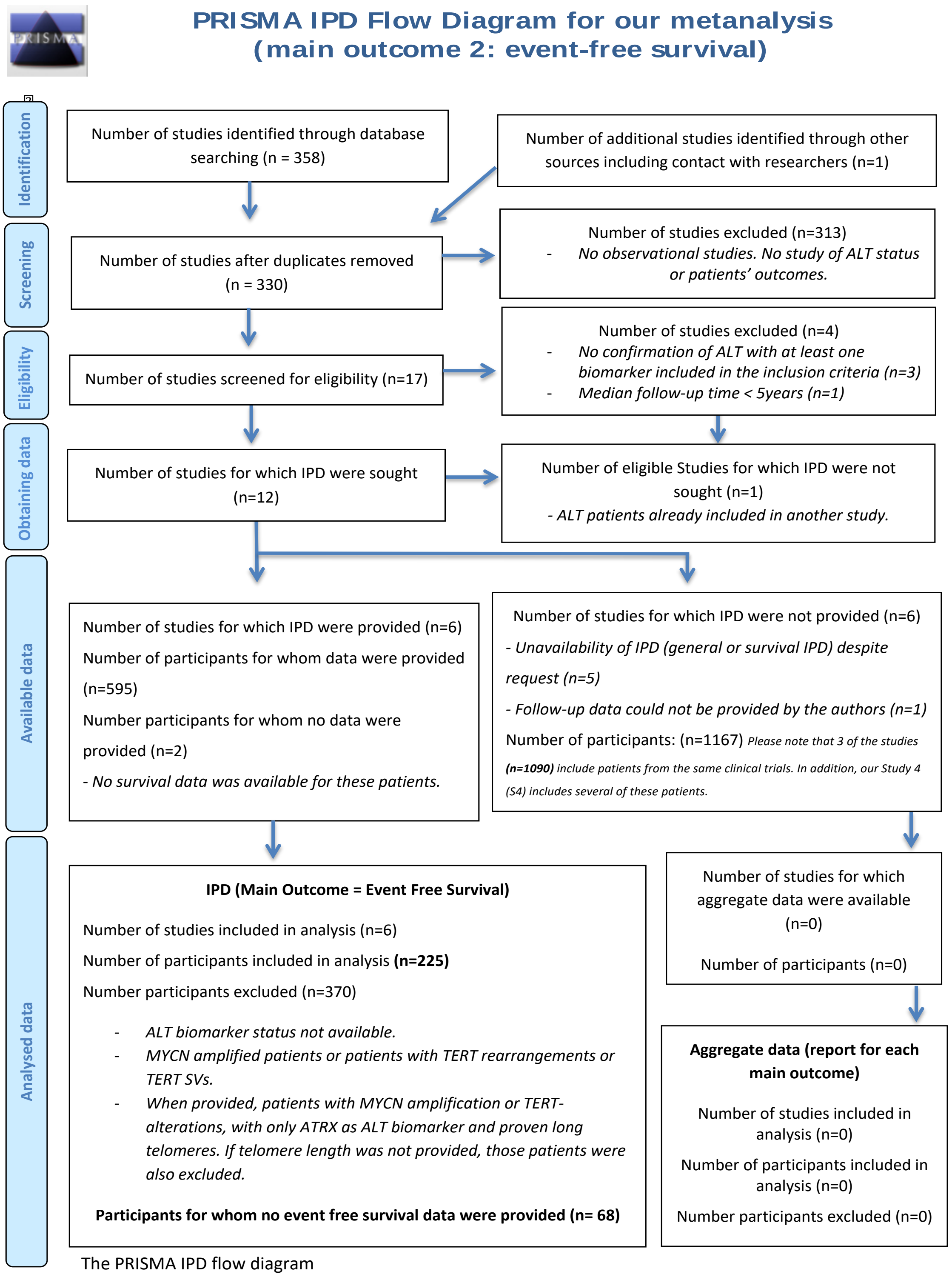

The PRISMA IPD flow diagram

© Reproduced with permission of the PRISMA IPD Group, which encourages sharing and reuse for non commercial purposes

Fig. S2. Prisma-IPD flow diagram from main outcome 2: event-free survival. A total of six studies and 225 patients were included in the IPD meta-analysis.

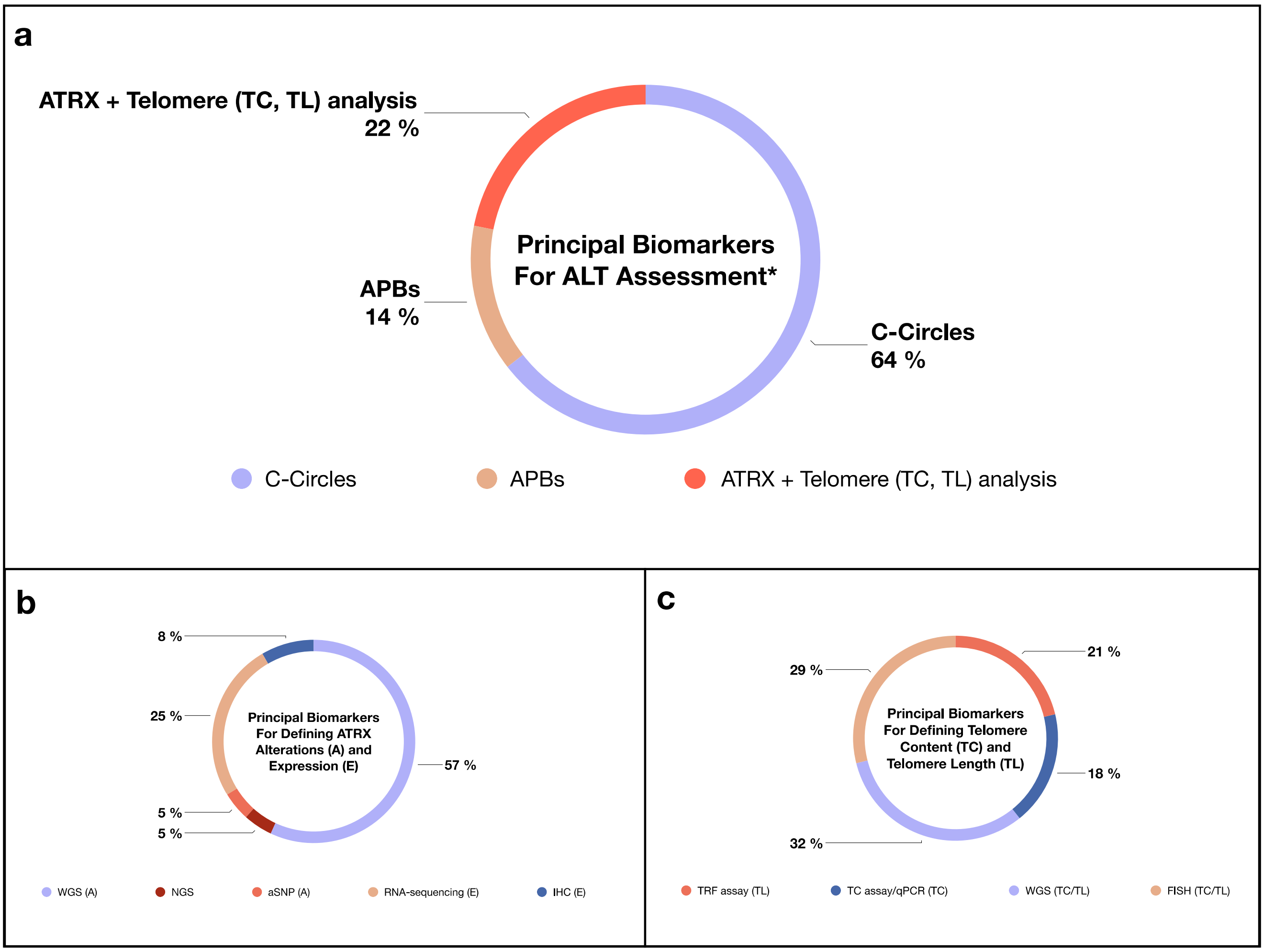

Fig. S3. *Principal biomarkers for ALT assessment in the Integrated Participant Cohort (IPC). (a) All studies analyzed telomere length (TL) and content (TC) and considered these in the classification process. However, for 22% of cases using ATRX as the principal biomarker, telomere analysis was essential in classifying tumors as ALT-positive. The majority of ALT-positive patients (64%) were classified primarily through the C-circle assay, with 14% identified via APBs. Refer to Figure 3, "Workflow for Research-Driven Assessment of ALT-positive Neuroblastoma," before interpreting this chart. **(b) Principal biomarkers for defining ATRX alterations (A) and expression (E) in the IPC.** NGS data was validated by Sanger sequencing. **WGS:** whole generation sequencing, **NGS:** next generation sequencing, **aSNP:** single nucleotide polymorphism array, **IHC:** immunohistochemistry. **(c) Principal biomarkers for defining telomere content (TC) and telomere length (TL) in the IPC. TRF:** telomere restriction fragment, **qPCR:** quantitative PCR, **WGS:** whole generation sequencing**, FISH:** fluorescent in situ hybridization.

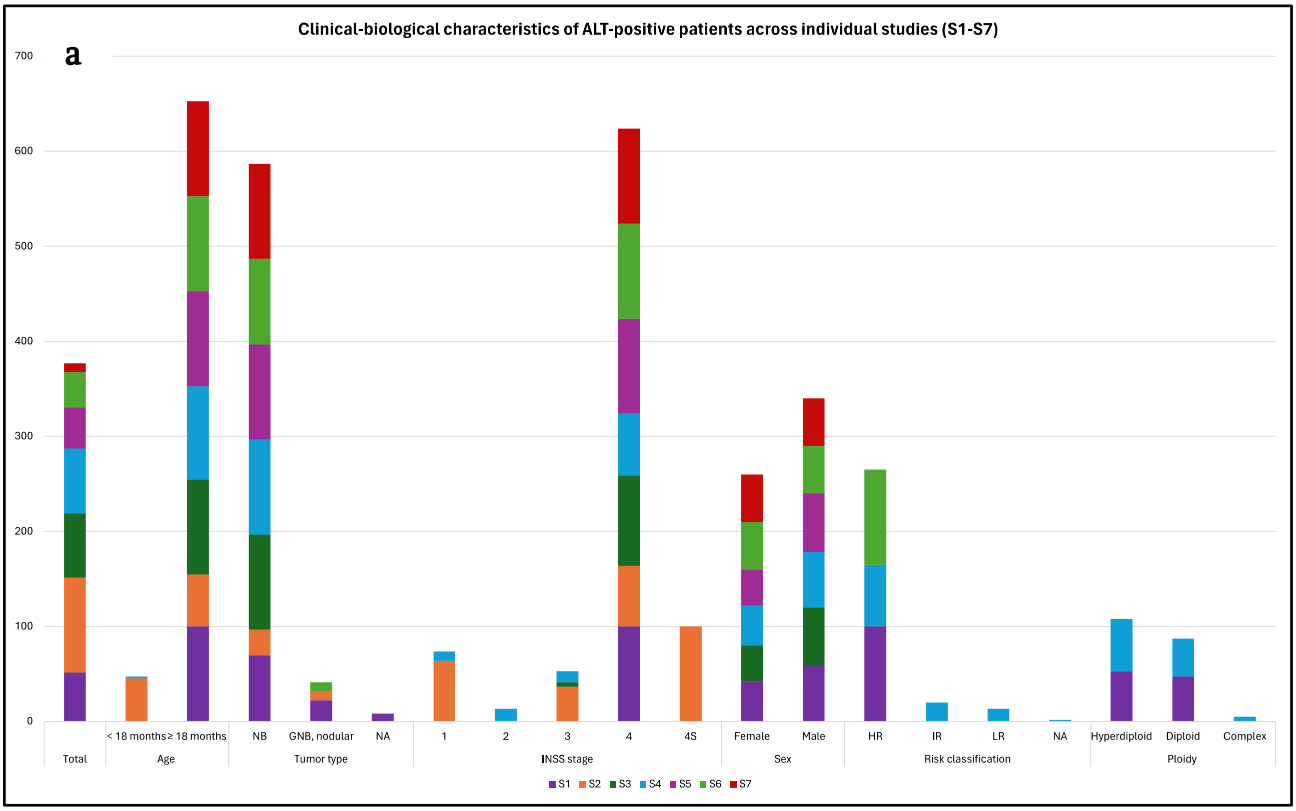

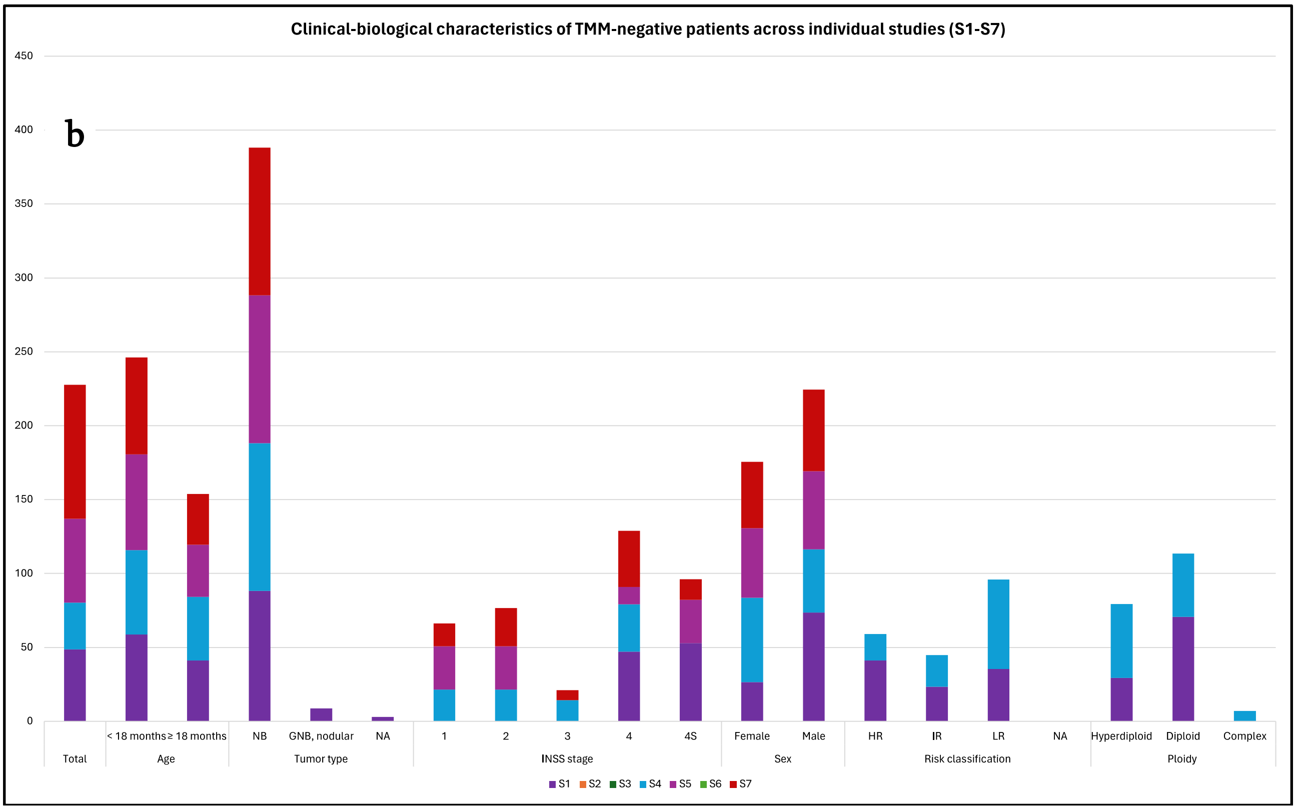

Fig. S4. Stacked bar plot assessing heterogeneity across studies (S1–S7). (a) Clinical-biological characteristics of ALT-positive patients across studies. All the seven studies analyzed, all provided data on ALT-positive patients. Notably, studies 1, 3, and 6 (ALTpos-n=67) exclusively enrolled high-risk or metastatic patients. **(b) Clinical-biological characteristics of TMM-negative patients across studies.** Within the TMM-negative sub-cohort, only studies 1, 4, 5, and 7 provided patients or adhered to our inclusion criteria. Here, a more balanced representation of subgroups is observed. Across both sub-cohorts, most patients (n=202) were diagnosed with neuroblastoma.

Supplementary tables

Table S1. (PDF) Prisma-IPD checklist.

Table S2. (Excel) This table lists patient ID, data availability, ALT status by biomarker, age, sex, INSS stage, risk classification, tumor type, ploidy, event-free and overall survival status, and follow-up times for patients forming both the ALT-positive and TMM-negative sub-cohorts.

a

Overall	ALT-positive		TMM-negative	
Study	5-year survival probability (95% CI)	10-year survival probability (95% CI)	5-year survival probability (95% CI)	10-year survival probability (95% CI)
S1	43% (29% - 64%)	16% (5.3% - 51%)	85% (73% - 98%)	77% (61% - 98%)
S2	25% (7.5% - 80%)	25% (7.5% - 80%)	-	-
S3	53% (35% - 81%)	25% (10% - 61%)	-	-
S4	77% (66% - 89%)	63% (50% -79%)	78% (64% - 95%)	78% (64% - 95%)
S5	54% (33% - 89%)	54% (33% - 89%)	94% (84% - 100%)	88% (73% - 100%)
S6	20% (5.8% - 69%)	20% (5.8% - 69%)	-	-
S7	0% (2.8% - 100%)	-	84% (75% - 95%)	84% (75% - 95%)
IPC	53% (45% - 62%)	40% (32% - 50%)	77% (71% - 84%)	72% (65% - 80%)
IPC Stage 4	48% (40% - 58%)	34% (26% - 45%)	61% (47% - 78%)	50% (35% - 72%)
IPC Non-stage 4	82% (67% - 100%)	73% (53% - 98%)	95% (91% - 100%)	95% (91% - 100%)

b

Event-free	ALT-positive		TMM-negative	
Study	5-year survival probability (95% CI)	10-year survival probability (95% CI)	5-year survival probability (95% CI)	10-year survival probability (95% CI)
S1	29% (17% - 50%)	25% (13% - 46%)	61% (46% - 80%)	61% (46% - 80%)
S2	-	-	-	-
S3	21% (8.7% - 49%)	21% (8.7% - 49%)	-	-
S4	20% (12% - 33%)	16% (8.9% - 29%)	61% (45% - 82%)	61% (45% - 82%)
S5	31% (14% - 70%)	23% (8.6% - 62%)	88% (74% - 100%)	88% (74% - 100%)
S6	10% (1.6% - 64%)	-	-	-
S7	-	-	-	-
IPC	21% (15% - 29%)	18% (12% - 26%)	67% (57% - 78%)	67% (57% - 78%)
IPC Stage 4	23% (16% - 32%)	20% (14% - 29%)	41% (26% - 64%)	41% (26% - 64%)
IPC Non-stage 4	10% (2.8% - 38%)	-	81% (70% - 92%)	81% (70% - 92%)

Table S3. (a) 5-year and 10-year overall survival probabilities across studies and in the IPC. Studies 2, 3 and 6 only included data on ALT-positive patients. **(b) 5-year and 10-year event-free survival probabilities across studies and in the IPC**. Event-free survival data was not available for S2. For S7, event-free survival was only available for ALT-positive patients. However, all patients included in S7 (n=6) were dead of disease before 5 years.

a

Overall (years)	ALT-positive	TMM-negative
Study	Median survival probability (95% CI)	Median survival probability (95% CI)
S1	4.6 (3.6, -)	-
S2	4.3 (3.1, -)	-
S3	5.4 (3.9, -)	-
S4	14 (11, -)	-
S5	- (1.8, -)	-
S6	3.6 (3.1, -)	-
S7	3.0 (2.7, -)	-
IPC	5.6 (4.5 - 9.7)	-
IPC Stage 4	4.8 (4.0 - 7.0)	7.3 (4.5, -)
IPC Non-stage 4	11 (11, -)	-

b

Event- free (years)	ALT-positive	TMM-negative
Study	Median survival probability (95% CI)	Median survival probability (95% CI)
S1	2.4 (1.8 - 4.8)	-
S2	-	-
S3	2.6 (1.3 - 3.6)	-
S4	1.8 (1.5 - 2.4)	-
S5	1.8 (1.5, -)	-
S6	1.6 (1.3, -)	-
S7	1.8 (1.2, -)	-
IPC	1.8 (1.8 - 2.3)	-
IPC Stage 4	1.9 (1.8 - 2.4)	2.4 (1.9, -)
IPC Non-stage 4	1.4 (0.90, 4.1)	-

Table S4. (a) Median overall survival probabilities across studies and in the IPC. Apart from the stage 4 sub-cohort, none of the TMM-negative subgroups across studies had survival rates lower than 50%, so median survival could not be calculated. **(b) Median event-free survival probabilities across studies and in the IPC**. The same applies for median event-free survival.

Supplementary data on individual studies

Tables presenting X-year survival probabilities and median survival times can be found in the previous section.

For the following tables S1, S4:

Study 1: Koneru B, Lopez G, Farooqi A, Conkrite KL, Nguyen TH, Macha SJ, et al. Telomere maintenance mechanisms define clinical outcome in high-risk neuroblastoma. Cancer Res. 2020;80(12):2663–75.

Clinical-biological features of patients in Study 1 (n=70)[a]								
		Total cohort		ALT positive		TMM negative		p-value[f]
		n[e]	%[e]	n[e]	%[e]	n[e]	%[e]	
Total		70		36	51,4	34	48,6	
Age[b]	< 18 months	20	28,6	0	0	20	58,8	8,60E-06
	≥ 18 months	50	71,4	36	100	14	41,2	
Tumor type	NB	55	78,6	25	69,4	30	88,2	
	GNB, nodular	11	15,7	8	22,2	3	8,8	
	NA	4	5,7	3	8,3	1	2,9	
INSS	Stage 4	52	74,3	36	100	16	47,1	9,50E-05
	Non-stage 4[c]	18	25,7	0	0	18	52,9	
Sex	Female	24	34,3	15	41,7	9	26,5	0.2771
	Male	46	65,7	21	58,3	25	73,5	
Risk classification[d]	HR	50	71,4	36	100	14	41,2	8,60E-06
	IR	8	11,4	0	0	8	23,5	
	LR	12	17,1	0	0	12	35,3	
	NA	0	0,0	0	0	0	0	
Ploidy	Hyperdiploid	29	41,4	19	52,8	10	29,4	0.0561
	Diploid	41	58,6	17	47,2	24	70,6	
	Complex	0	0,0	0	0	0	0	
ATRX status	Altered	26	37,1	26	72,2	0	0	
	Wildtype	10	14,3	10	27,8	0	0	

[a] n refers to the number of patients.
[b] Age at diagnosis.
[c] Non-stage 4 includes: **stage 1**, **stage 2** , **stage 3** and **stage 4S**.
[d] Risk classification abbreviations: **HR** = high-risk, **IR** = intermediate-risk, **LR** = low-risk, and **NA** = not available.
[e] Values are depicted both in terms of absolute patient counts (n) and as proportions (%) relative to the total number of patients within each group (total cohort, ALT positive, TMM negative).
[f] P values were calculated using Chi-squared and Fisher's exact tests.

Table S5. Clinical-biological characteristics of patients in S1. S1 included a total of 70 patients. The ALT-positive sub-cohort constituted 51.4% of patients (n=36), while the TMM-negative sub-cohort accounted for 48.6% (n=34) of total patients. Concerning age, 100% of patients in the ALT-positive sub-cohort were diagnosed at 18 months or older, contrasting with 58.8% of patients in the TMM-negative sub-cohort who were under 18 months at diagnosis. Differences between sexes were not significant within the two sub-cohorts (p=0.2771) and no significant differences were found in terms of ploidy. Among the 70 patients, 55 were diagnosed with neuroblastoma, with only 11 diagnosed with nodular ganglioneuroblastoma, the majority of whom were ALT-positive (n=8). S1 included only metastatic patients (stage 4 and 4S), meaning that 100% of ALT-positive patients were diagnosed at stage 4 according to INSS criteria, whereas 52.9% of the TMM-negative patients were diagnosed at stage 4S. Similarly, 100% of ALT-positive patients were designated as high-risk, while 58.8% of the TMM-negative sub-cohort were classified as non-high risk. Moreover, 72.2% of the ALT-positive patients had ATRX alterations. [c] Non-stage 4 includes: stage 1 (ALTposn = 0, TMMnegn = 0), stage 2 (ALTposn = 0, TMMnegn = 0), stage 3 (ALTposn = 0, TMMnegn = 0) and stage 4S (ALTposn = 0, TMMnegn = 18).

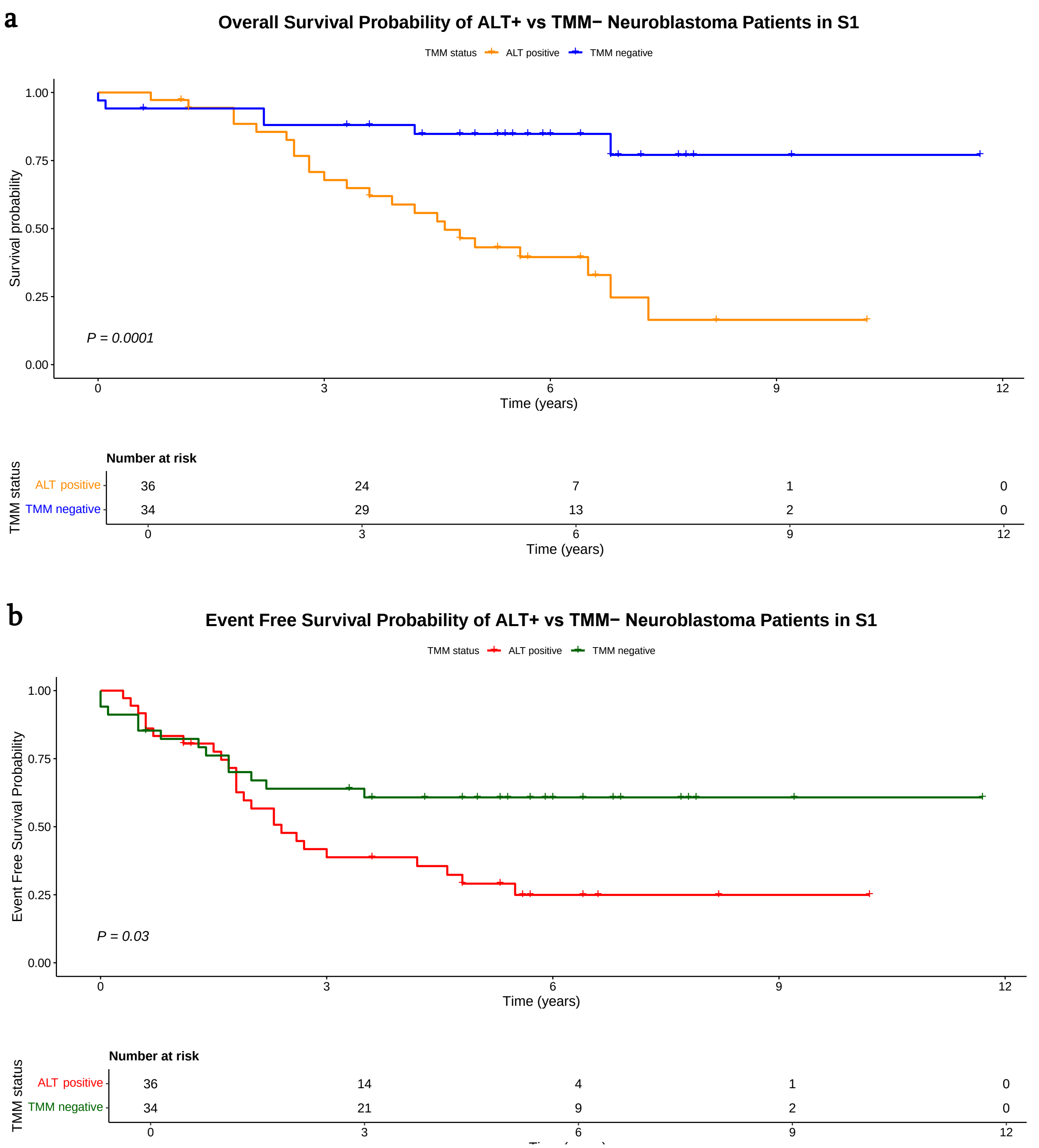

Fig. S5. (a) Overall survival probability in S1. Kaplan-Meier analysis in overall survival revealed significantly distinct survival outcomes in the ALT-positive and TMM-negative sub-cohorts in S1 (p = 0.0001). The 5-year and 10-year overall survival probabilities varied across subgroups, with the ALT-positive sub-cohort showing a significantly lower 5-year overall survival probability of 43% compared to 81% in the TMM-negative sub-cohort. Importantly, the overall survival probability of the ALT-positive sub-cohort decreased to 10% at 10 years, while in the TMM-negative sub-cohort it remained above 70%. Median survival in the ALT-positive sub-cohort was 4.6 years. **(b) Event-free survival probability in S1**. ALT-positive patients had a significantly shorter event-free survival than TMM-negative patients. The 5-year event-free survival probability was 29% in the ALT-positive subgroup compared to 61% in the TMM-negative subgroup. Importantly, while the event-free survival probability of the TMM-negative subgroup remained the same at 10 years, in the ALT-positive subgroup it decreased to 25%, with a median event-free survival time of 2.4 years.

Study 2: Kurihara S, Hiyama E, Onitake Y, Yamaoka E, Hiyama K. Clinical features of ATRX or DAXX mutated neuroblastoma. J Pediatr Surg. 2014;49(12):1835–8.

Clinical-biological features of patients in Study 2 (n=11)[a]			
		ALT positive	
		n[d]	%[d]
**Total**		**11**	**100**
**Age[b]**	**< 18 months**	5	45,5
	**≥ 18 months**	6	54,5
**INSS**	**Stage 4**	7	63,6
	**Non-stage 4[c]**	3	36,4
**Sex**	**Female**	4	36,4
	**Male**	7	63,6
**ATRX status**	**Altered**	11	100
	**Wildtype**	0	0

[a] n refers to the number of patients.
[b] Age at diagnosis.
[c] Non-stage 4 includes: **stage 1** ($ALTpos_n$ = 1), **stage 2** ($ALTpos_n$ = 2), **stage 3** ($ALTpos_n$ = 0) and **stage 4S** ($ALTpos_n$ = 0).
[d] Values are depicted both in terms of absolute patient counts (n) and as proportions (%) relative to the total number of patients within each group (total cohort, ALT positive, TMM negative).

Table S6. Clinical-biological characteristics of patients in S2. S2 yielded only ALT-positive patients (n=11). Concerning age, 54.5% of patients were diagnosed at 18 months or older, contrasting with 45.5% of patients who were younger than 18 months at diagnosis. All patients were diagnosed with neuroblastoma. Notably, 63.6% of patients were diagnosed at stage 4, compared with 36.4% as non-stage 4 according to INSS criteria. Also noteworthy is the fact that all ALT-positive patients exhibited ATRX alterations. However, since ATRX was the primary marker used for detection, the ALT-positive patients might be underrepresented in this study.

a

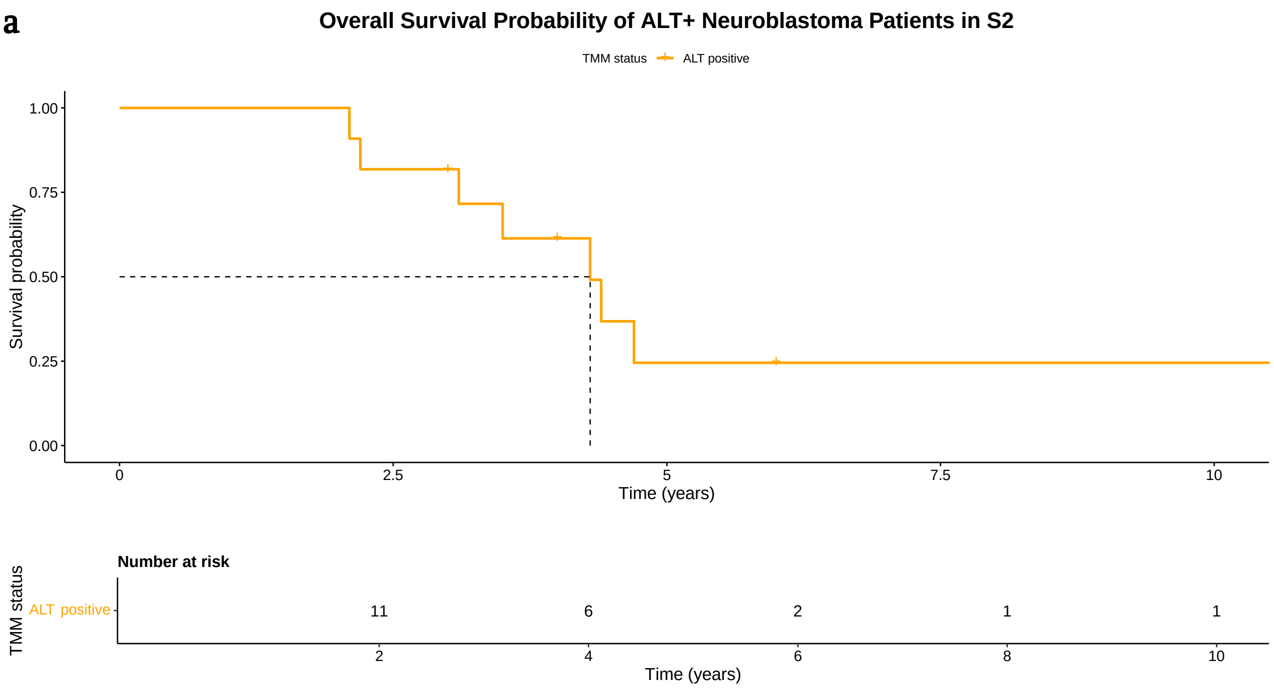

Fig. S6. Overall survival probability in S2. Kaplan-Meier analysis in overall survival revealed a 5-year overall survival probability of 25%. Due to the small sample size, no differences at 10 years were observed. Median survival of the ALT-positive patients was 4.3 years.

Study 3: Cheung N-KV. Association of age at diagnosis and genetic mutations in patients with neuroblastoma. JAMA. 2012;307(10):1062.

Clinical-biological features of patients in Study 3 (n=21)[a]			
		ALT positive	
		n[d]	%[d]
**Total**		**21**	**67,7**
**Age[b]**	**< 18 months**	0	0
	**≥ 18 months**	21	100
**Tumor type**	**NB**	21	100
	**GNB, nodular**	0	0
	**NA**	0	0
**INSS**	**Stage 4**	20	95,2
	**Non-stage 4[c]**	1	4,8
**Sex**	**Female**	8	38,1
	**Male**	13	61,9
**ATRX status**	**Altered**	21	100
	**Wildtype**	0	0

[a] n refers to the number of patients.
[b] Age at diagnosis.
[c] Non-stage 4 includes: **stage 1** (ALTposn = 0), **stage 2** (ALTposn = 0), **stage 3** (ALTposn = 1) and **stage 4S** (ALTposn = 0).
[d] Values are depicted both in terms of absolute patient counts (n) and as proportions (%) relative to the total number of patients within each group (total cohort, ALT positive, TMM negative).

Table S7. Clinical-biological characteristics of patients in S3. In S3 only ALT-positive patients met our selection criteria (n=21). Concerning age, all patients were diagnosed at 18 months or older and all patients were diagnosed with neuroblastoma. S3 only included high-risk and metastatic patients, thus 95.2% of ALT-positive patients were diagnosed at stage 4 according to INSS criteria. Also is noteworthy the fact that all ALT-positive patients exhibited ATRX alterations. However, since ATRX was the primary marker used for detection, the ALT-positive patients might be underrepresented in this study.

a

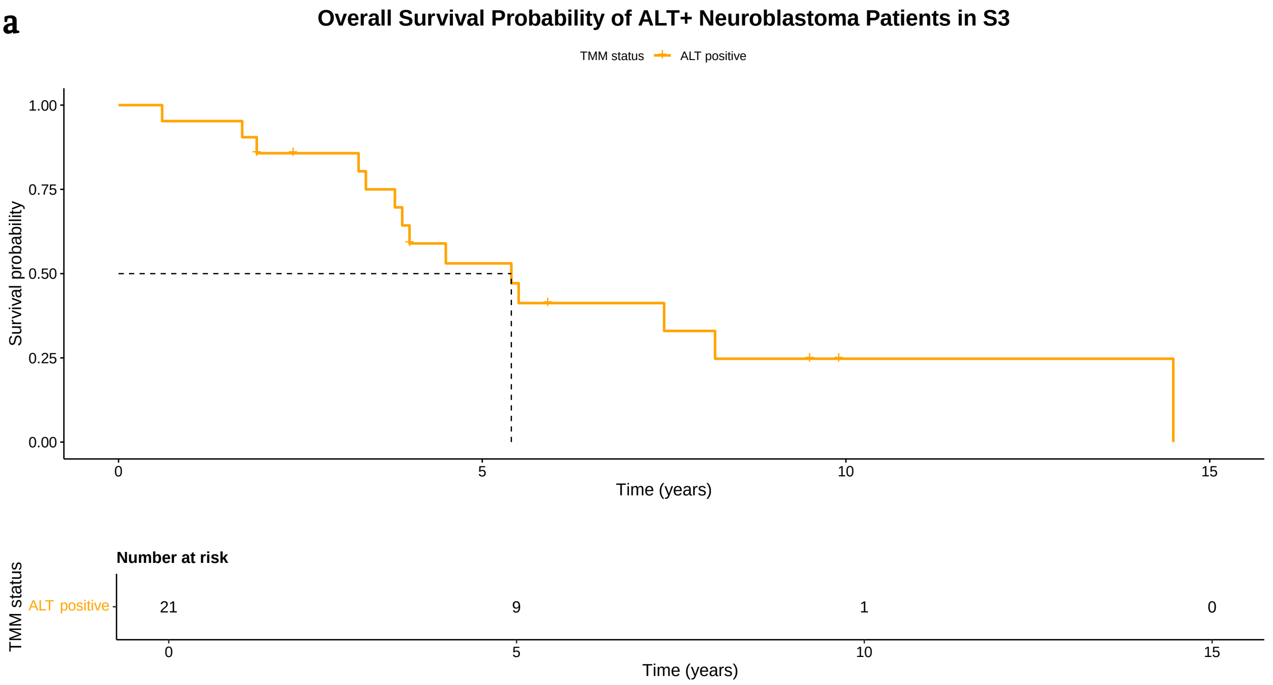

b

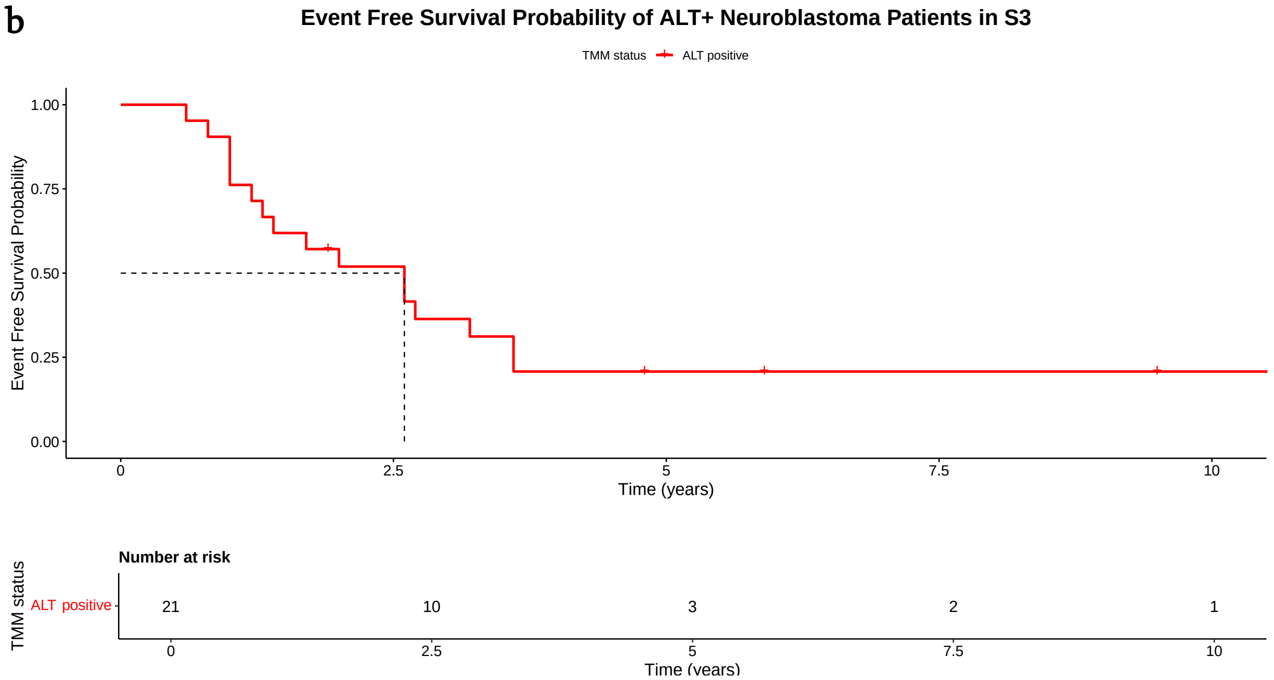

Fig. S7. (a) Overall survival probability in S3. Kaplan-Meier analysis in overall survival revealed a 5-year overall survival probability of 53%, decreasing to 25% at 10 years. The median survival of the ALT-positive patients was 5.4 years. **(b) Event-free survival probability in S3.** Kaplan-Meier analysis in event-free survival revealed a 5-year and 10-year event-free survival probability of 21%, while the median event-free survival of the ALT-positive patients was 2.6 years.

Study 4: Hartlieb SA, Sieverling L, Nadler-Holly M, Ziehm M, Toprak UH, Herrmann C, et al. Alternative lengthening of telomeres in childhood neuroblastoma from genome to proteome. Nat Commun. 2021;12(1).

Clinical-biological features of patients in Study 4 (n=88)[a]									
		Total cohort		ALT positive		TMM negative		p-value[f]
		n[e]	%[e]	n[e]	%[e]	n[e]	%[e]	
Total		88		60	68,2	28	31,8	
Age[b]	< 18 months	17	19,3	1	1,7	16	57,1	3,01E-06
	≥ 18 months	71	80,7	59	98,3	12	42,9	
Tumor type	NB	88	100	60	100	28	100	
	GNB, nodular	0	0	0	0	0	0	
	NA	0	0	0	0	0	0	
INSS	Stage 4	48	54,5	39	65	9	32,1	0.009367
	Non-stage 4[c]	40	45,5	21	35	19	67,9	
Sex	Female	41	46,6	25	41,7	16	57,1	0.2601
	Male	47	53,4	35	58,3	12	42,9	
Risk classification[d]	HR	44	50,0	39	65	5	17,9	7,40E-03
	IR	18	20,5	12	20	6	21,4	
	LR	25	28,4	8	13,3	17	60,7	
	NA	1	1,1	1	1,7	0	0	
Ploidy	Hyperdiploid	47	53,4	33	55	14	50	0.8121
	Diploid	36	40,9	24	40	12	42,9	
	Complex	5	5,7	3	5	2	7,1	
ATRX status	Altered	33	37,5	33	55	0	0	
	Wildtype	27	30,7	27	45	0	0	

Table S8. Clinical-biological characteristics of patients in S4. S4 included a total of 88 patients. The ALT-positive sub-cohort constituted 68.2% of patients (n=60), while the TMM-negative sub-cohort accounted for 31.8% (n=28) of total patients. Concerning age, 98.3% of patients in the ALT-positive sub-cohort were diagnosed at 18 months or older, contrasting with 57.1% of patients in the TMM-negative sub-cohort who were under 18 months at diagnosis. Differences between sexes were not significant within the two sub-cohorts (p=0.2601) and no significant differences were found in terms of ploidy. All patients were diagnosed with neuroblastoma. Notably, 35% of ALT-positive patients compared with 67.9% of TMM-negative patients were diagnosed as non-stage 4 according to INSS criteria. Similarly, 65% of ALT-positive patients were designated as high-risk, while 82.1% of the TMM-negative sub-cohort were classified as non-high risk. Moreover, 55% of the ALT-positive patients had ATRX alterations. [c] Non-stage 4 includes: stage 1 (ALTposn = 6, TMMnegn = 6), stage 2 (ALTposn = 8, TMMnegn = 6), stage 3 (ALTposn = 7, TMMnegn = 4) and stage 4S (ALTposn = 0, TMMnegn = 3).

a

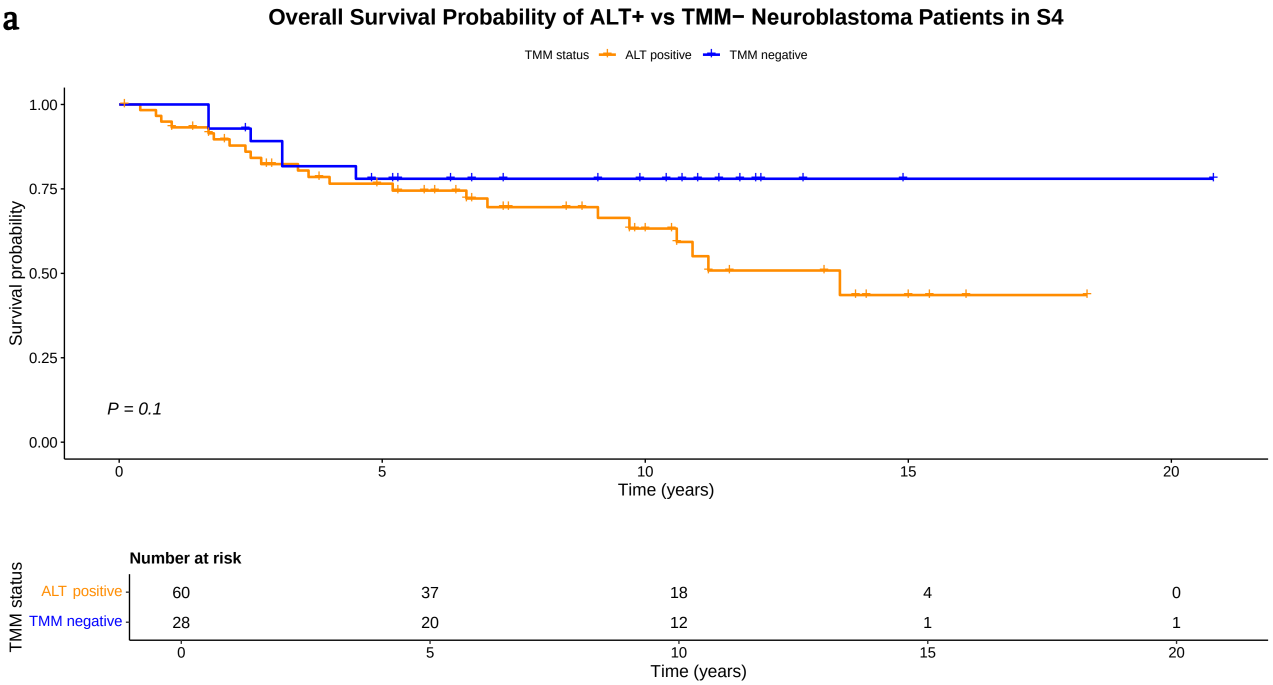

b

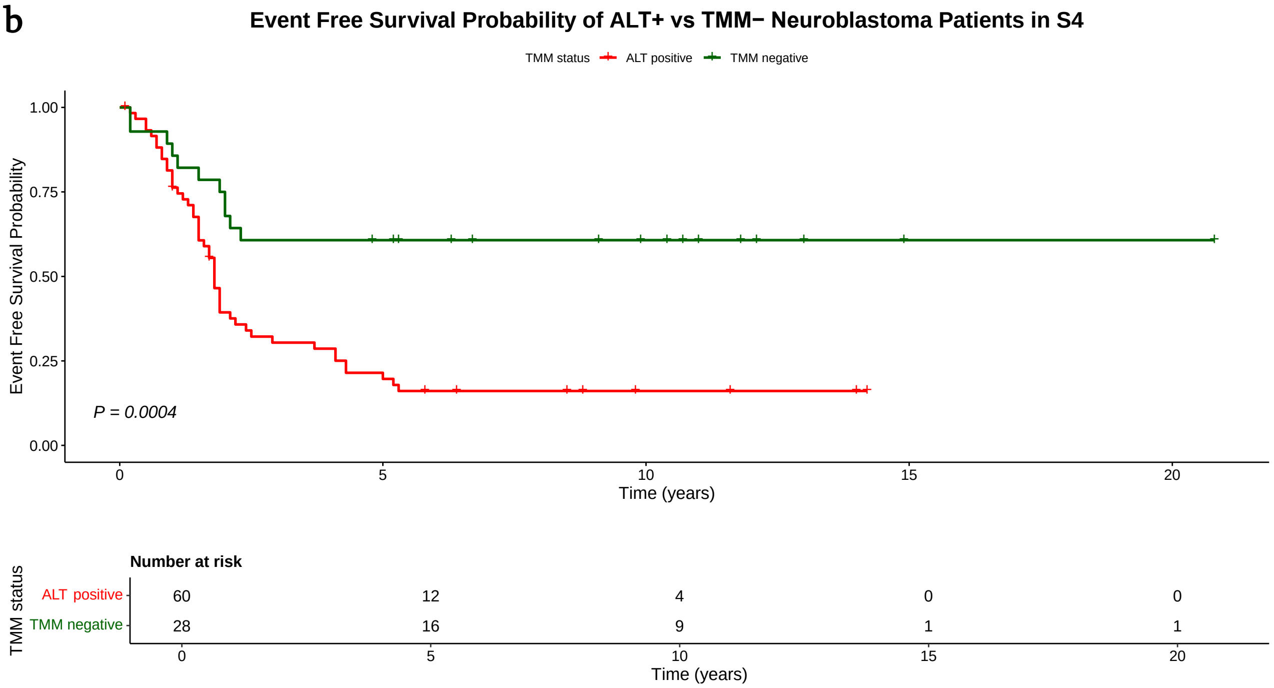

Fig. S8. (a) Overall survival probability in S4. Kaplan-Meier analysis of overall survival in S4 revealed discernible survival trends between the ALT-positive and TMM-negative sub-cohorts, although statistical significance was not reached (p = 0.1). The ALT-positive subgroup exhibited a 5-year overall survival probability of 77%, which closely paralleled the 78% observed in the TMM-negative subgroup. However, the overall survival probability of the ALT-positive sub-cohort decreased to 63% at 10-years, while in the TMM-negative sub-cohort it remained above 70%. Median survival in the ALT-positive sub-cohort was 14 years. **(b) Event-free survival probability in S4**. ALT-positive patients had a significantly shorter event-free survival than TMM-negative patients (p=0.0004). The 5-year event-free survival probability in the ALT-positive subgroup was 20% compared to 61% in the TMM-negative subgroup. Importantly, while the event-free survival probability of the TMM-negative subgroup remained the same at 10 years, in the ALT-positive subgroup it decreased to 16%, with a median event-free survival time of 1.8 years.

Study 5: Meeser A, Bartenhagen C, Werr L, Hellmann A-M, Kahlert Y, Hemstedt N, et al. Reliable assessment of telomere maintenance mechanisms in neuroblastoma. Cell Biosci. 2022;12(1).

Clinical-biological features of patients in Study 5 (n=30)[a]									
		Total cohort		ALT positive		TMM negative		p-value[e]
		n[d]	%[d]	n[d]	%[d]	n[d]	%[d]	
**Total**		**30**		**13**	**43,3**	**17**	**56,7**	
**Age[b]**	**< 18 months**	11	36,7	0	0	11	64,7	**0.000317**
	**≥ 18 months**	19	63,3	13	100	6	35,3	
**Tumor type**	**NB**	30	100	13	100	17	100	
	**GNB, nodular**	0	0	0	0	0	0	
	**NA**	0	0	0	0	0	0	
**INSS**	**Stage 4**	15	50	13	100	2	11,8	**1,75E-03**
	**Non-stage 4[c]**	15	50	0	0	15	88,2	
**Sex**	**Female**	13	43,3	5	38,5	8	47,1	**0.921**
	**Male**	17	56,7	8	61,5	9	52,9	

[a] n refers to the number of patients.
[b] Age at diagnosis.
[c] Non-stage 4 includes: **stage 1** ($ALTpos_n$ = 0, $TMMneg_n$ = 5), **stage 2** ($ALTpos_n$ = 0, $TMMneg_n$ = 5), **stage 3** ($ALTpos_n$ = 0, $TMMneg_n$ = 0) and **stage 4S** ($ALTpos_n$ = 0, $TMMneg_n$ = 5).
[d] Values are depicted both in terms of absolute patient counts (n) and as proportions (%) relative to the total number of patients within each group (total cohort, ALT positive, TMM negative).
[e] P values were calculated using Chi-squared and Fisher's exact tests.

Table S9. Clinical-biological characteristics of patients in S5. S5 included a total of 30 patients. The ALT-positive sub-cohort constituted 43.3% of patients (n=13), while the TMM-negative sub-cohort accounted for 56.7% (n=17). Concerning age, 100% of patients in the ALT-positive sub-cohort were diagnosed at 18 months or older, contrasting with 64.7% of patients in the TMM-negative sub-cohort who were under 18 months at diagnosis. Differences between sexes were not significant within the two sub-cohorts (p=0.921). All patients were diagnosed with neuroblastoma. Notably, 100% of ALT-positive patients were diagnosed at stage 4 according to INSS criteria, whereas 88.2% of the TMM-negative patients were diagnosed as non-stage 4.

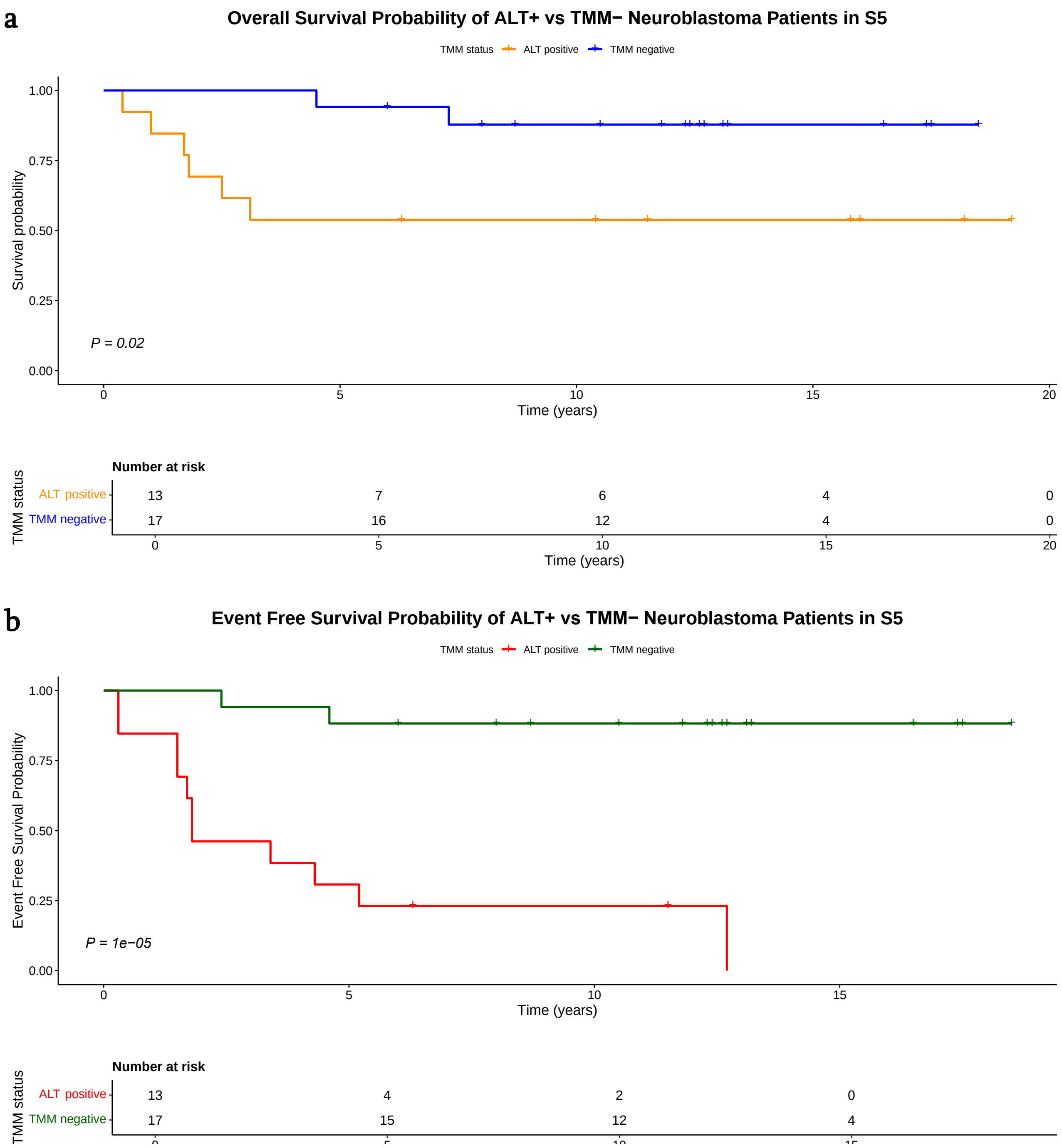

Fig. S9. (a) Overall survival probability in S5. Kaplan-Meier analysis in overall survival revealed significantly different survival outcomes between the ALT-positive and TMM-negative sub-cohorts in S1 (p = 0.02). The 5-year and 10-year overall survival probabilities varied across subgroups, with the ALT-positive sub-cohort showing a significantly lower 5-year overall survival probability of 54% compared to 94% in the TMM-negative sub-cohort. No 10-year differences were observed in this study, probably due to the small sample size. The overall survival probability of the TMM-negative sub-cohort remained above 80% at 10-years. Median survival could not be calculated. **(b) Event-free survival probability in S5**. ALT-positive patients had significantly shorter event-free survival than TMM-negative patients (p=1e-05). The 5-year event-free survival probability in the ALT-positive subgroup was 31%, compared to the 88% in the TMM-negative subgroup. Importantly, while the event-free survival probability of the TMM-negative subgroup remained the same at 10 years, in the ALT-positive subgroup it decreased to 23%, with a median event-free survival time of 1.8 years.

Study 6: Lundberg G, Sehic D, Länsberg J-K, Øra I, Frigyesi A, Castel V, et al. Alternative lengthening of telomeres—An enhanced chromosomal instability in aggressive non-*MYCN* amplified and telomere elongated neuroblastomas. Genes Chromosomes Cancer. 2011;50(4):250–62.

Clinical-biological features of patients in Study 6 (n=10)[a]			
		ALT positive	
		n[e]	%[e]
**Total**		**10**	**37**
**Age[b]**	**< 18 months**	0	0
	**≥ 18 months**	10	100
**Tumor type**	**NB**	9	90
	**GNB, nodular**	1	10
	**NA**	0	0
**INSS**	**Stage 4**	10	100
	**Non-stage 4[c]**	0	0
**Sex**	**Female**	5	50
	**Male**	5	50
**Risk classification[d]**	**HR**	10	100
	**IR**	0	0
	**LR**	0	0
	**NA**	0	0

[a] n refers to the number of patients.
[b] Age at diagnosis.
[c] Non-stage 4 includes: stage 1 ($ALTpos_n$ = 0), stage 2 ($ALTpos_n$ = 0), stage 3 ($ALTpos_n$ = 0) and stage 4S ($ALTpos_n$ = 0).
[d] Risk classification abbreviations: **HR** = high-risk, **IR** = intermediate-risk, **LR** = low-risk, and **NA** = not available.
[e] Values are depicted both in terms of absolute patient counts (n) and as proportions (%) relative to the total number of patients within each group (total cohort, ALT positive, TMM negative).

Table S10. Clinical-biological characteristics of patients in S6. In S6 only ALT-positive patients met our selection criteria (n=10). Concerning age, all patients were diagnosed at 18 months or older and all patients but one were diagnosed with neuroblastoma. All patients were also diagnosed at stage 4 according to INSS criteria and classified as high-risk.

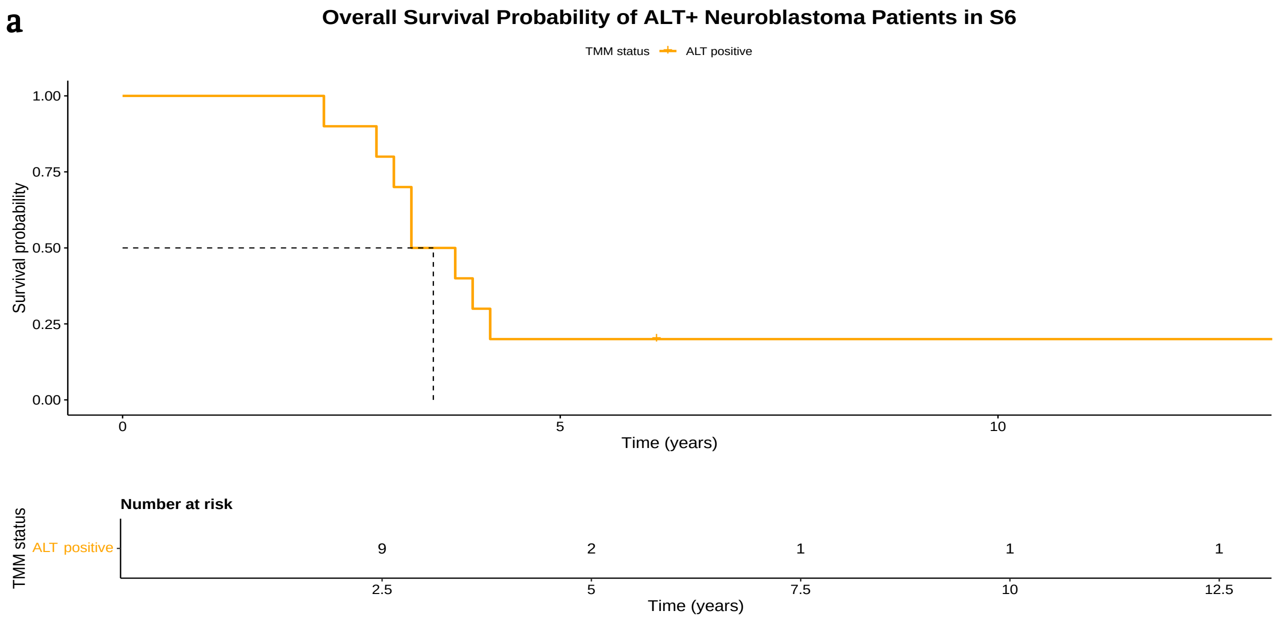

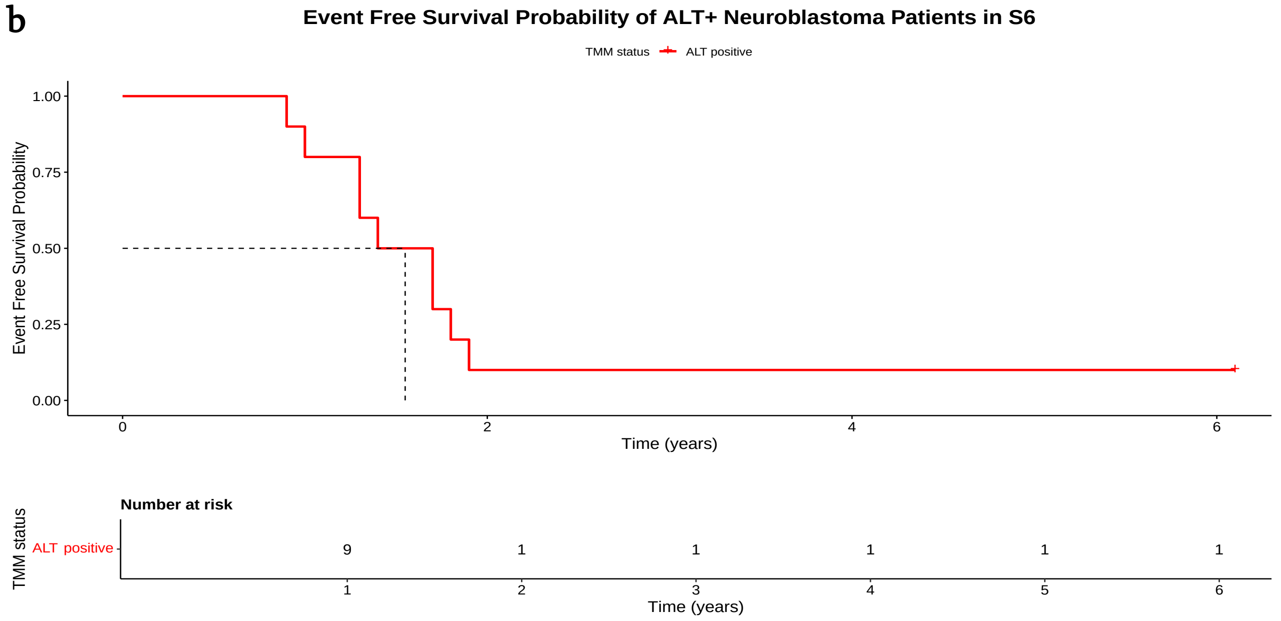

Fig. S10. (a) Overall survival probability in S6. Kaplan-Meier analysis in overall survival revealed a 5-year overall survival probability of 20%. Due to the small sample size, no differences at 10 years were observed. In addition, median survival of the ALT-positive patients was 3.6 years. **(b) Event-free survival probability in S6.** Kaplan-Meier analysis in event-free survival revealed a 5-year event-free survival probability of 10%. In addition, median event-free survival in the ALT-positive patients was 1.6 years.

Study 7: Valentijn LJ, Koster J, Zwijnenburg DA, Hasselt NE, van Sluis P, Volckmann R, et al. TERT rearrangements are frequent in neuroblastoma and identify aggressive tumors. Nat Genet. 2015;47(12):1411–4.

Survival data from ALT-positive patients were updated in Study 8: van Gerven MR, Bozsaky E, Matser YAH, Vosseberg J, Taschner-Mandl S, Koster J, et al. Mutational spectrum of *ATRX* aberrations in neuroblastoma and associated patient and tumor characteristics. Cancer Sci. 2022;113(6):2167–78.

To enhance clarity and facilitate data analysis, both studies were combined into Study 7 in the supplementary data.

Clinical-biological features of patients in Study 7 (n=64)[a]

		Total cohort		ALT positive		TMM negative		p-value[e]
		n[d]	%[d]	n[d]	%[d]	n[d]	%[d]	
**Total**		**64**		**6**	**9,4**	**58**	**90,6**	
**Age[b]**	**< 18 months**	38	59,4	0	0	38	65,5	**0.003071**
	**≥ 18 months**	26	40,6	6	100	20	34,5	
**Tumor type**	**NB**	64	100	6	100	58	100	
	**GNB, nodular**	0	0	0	0	0	0	
	**NA**	0	0	0	0	0	0	
**INSS**	**Stage 4**	28	43,8	6	100	22	37,9	**0.1369**
	**Non-stage 4[c]**	36	56,3	0	0	36	62,1	
**Sex**	**Female**	29	45,3	3	50	26	44,8	**1**
	**Male**	35	54,7	3	50	32	55,2	
**ATRX status**	**Altered**	6	9,4	6	100	0	0	
	**Wildtype**	0	0	0	0	0	0	

[a] n refers to the number of patients.
[b] Age at diagnosis.
[c] Non-stage 4 includes: **stage 1** ($ALTpos_n$ = 9, $TMMneg_n$ = 9), **stage 2** ($ALTpos_n$ = 0, $TMMneg_n$ = 15), **stage 3** ($ALTpos_n$ = 0, $TMMneg_n$ = 4) and **stage 4S** ($ALTpos_n$ = 0, $TMMneg_n$ = 8).
[d] Values are depicted both in terms of absolute patient counts (n) and as proportions (%) relative to the total number of patients within each group (total cohort, ALT positive, TMM negative).
[e] P values were calculated using Chi-squared and Fisher's exact tests.

Table S11. Clinical-biological characteristics of patients in S7. S7 included a total of 64 patients. The ALT-positive sub-cohort constituted 9.4% of patients (n=6), while the TMM-negative sub-cohort accounted for 90.6% (n=58) of the total patients. Concerning age, 100% of patients in the ALT-positive sub-cohort were diagnosed at 18 months or older, contrasting with 65.5% of patients in the TMM-negative sub-cohort, who were younger than 18 months at diagnosis. Differences between sexes were not significant within the two sub-cohorts (p=1). All patients were diagnosed with neuroblastoma. Additionally, 100% of ALT-positive patients were diagnosed at stage 4 according to INSS criteria, whereas 62.1% of the TMM-negative patients were diagnosed as non-stage 4. Furthermore, it's noteworthy that all ALT-positive patients exhibited ATRX alterations. However, since ATRX was the primary marker used for detection, the ALT-positive sub-cohort might be underrepresented in this study.

a

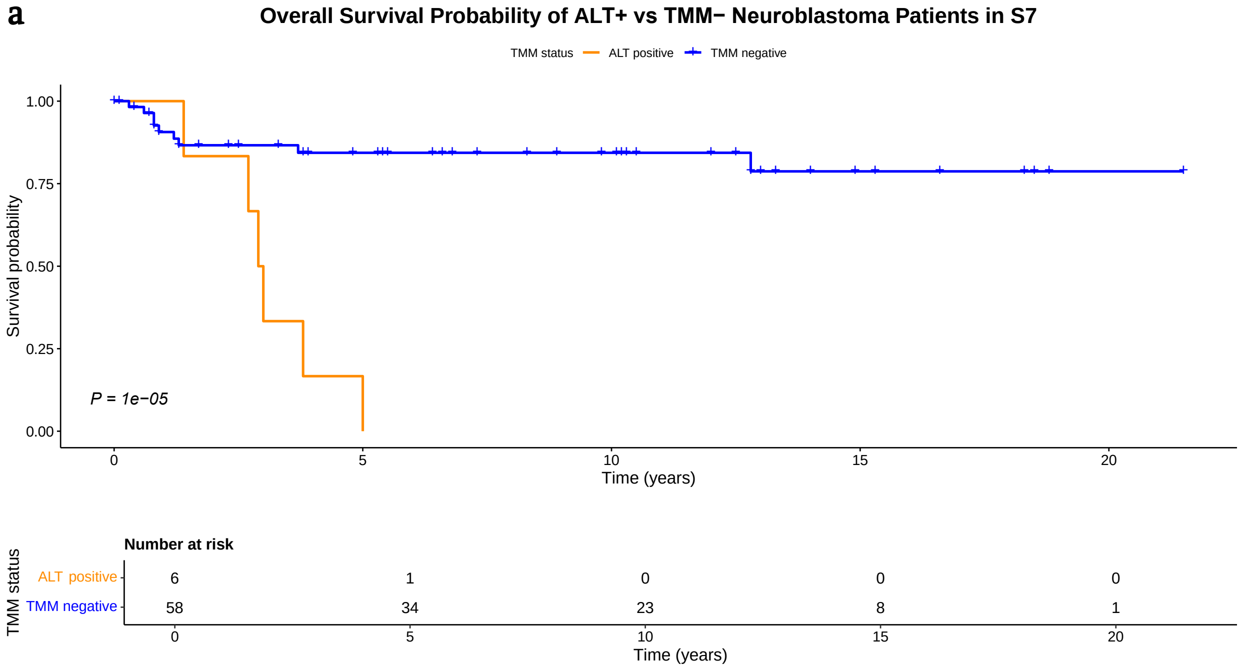

b

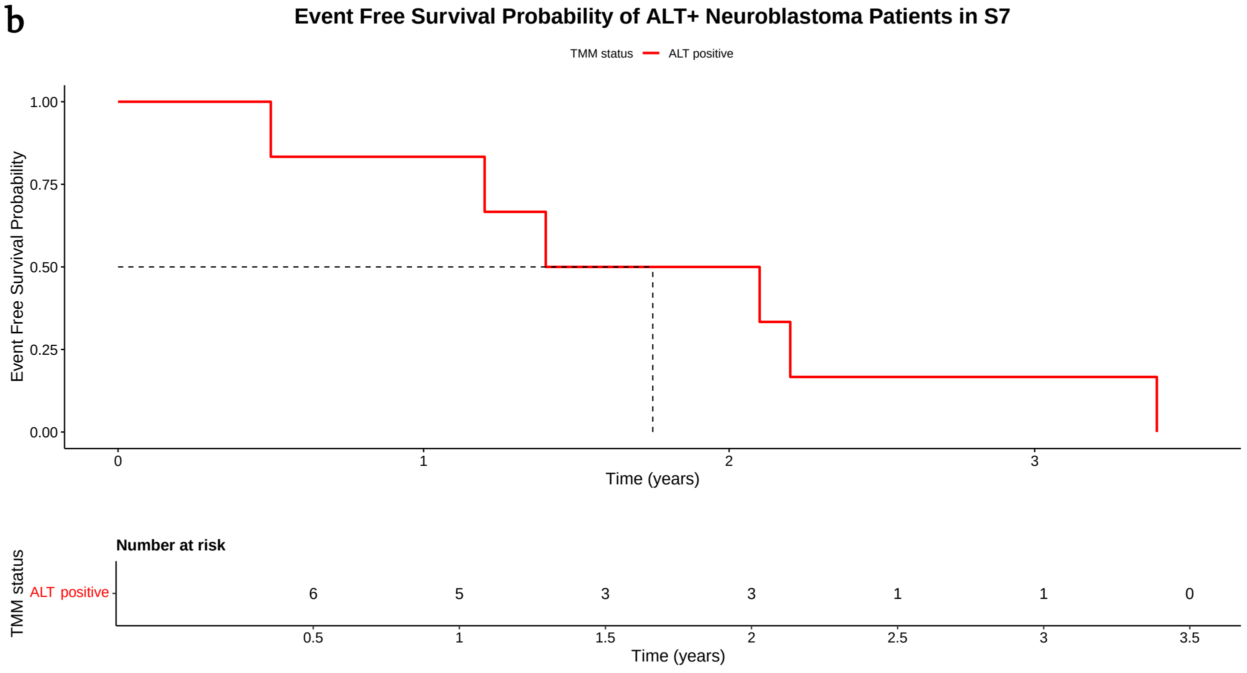

Fig. S11. (a) Overall survival probability in S7. Kaplan-Meier analysis in overall survival revealed significantly different survival outcomes between the ALT-positive and TMM-negative sub-cohorts in S7 (p = 1e-05). The 5-year and 10-year overall survival probabilities varied across subgroups, with the ALT-positive sub-cohort showing a significantly lower 5-year overall survival probability of 0% compared to 84% in the TMM-negative sub-cohort. However, it is important to note that the ALT-positive subgroup consisted of only 6 patients. The overall survival probability of the TMM-negative sub-cohort remained above 80% at 10 years. Median survival of the ALT-positive patients was 3.0 years. **(b) Event-free survival probability in S5**. Kaplan-Meier analysis in event-free survival could not provide a 5-year and 10-year event-free survival probability as all patients were dead of disease after 2 years. Median event-free survival in the ALT-positive patients was 1.8 years.

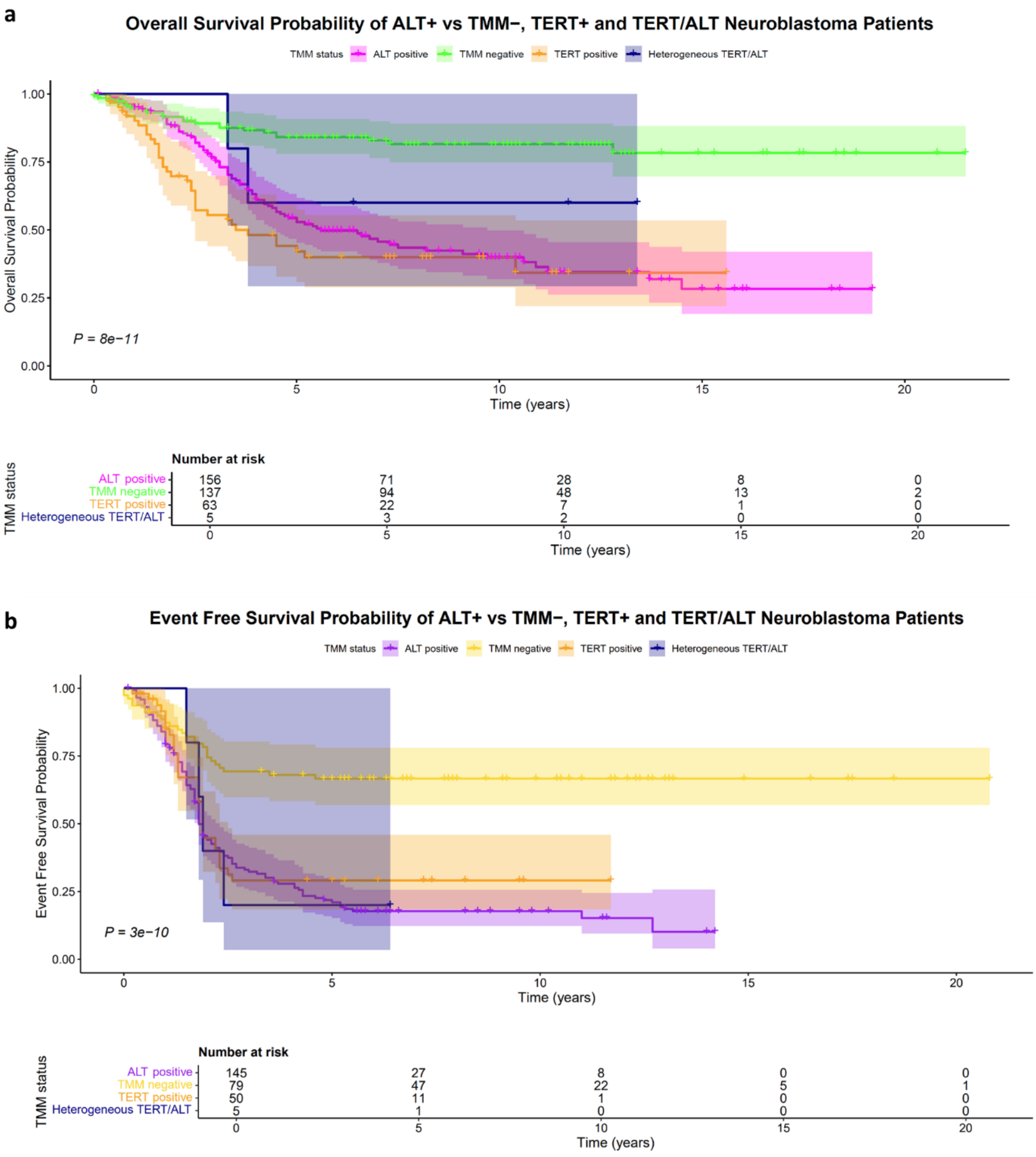

Fig. S12. Survival probabilities among ALT-positive, TMM-negative, TERT-positive and TERT/ ALT heterogeneous patients. (a) Overall survival probabilities of the sub-cohorts. Kaplan-Meier analysis of overall survival revealed significantly distinct outcomes among the ALT-positive, TMM-negative, TERT-positive, and heterogeneous TERT/ALT sub-cohorts ($p < 0.001$). At 5 years, the overall survival (OS) probability was 42% for the TERT-positive subgroup, and 53% for the ALT-positive subgroup, compared to 77% in the TMM-negative sub-cohort and, interestingly, 60% for the heterogeneous TERT/ALT subgroup. Pivotally, by 10 years, the OS probabilities for both the ALT-positive and TERT-positive subgroups had declined to 40%, while in the TMM-negative sub-cohort it remained above 70%. However, data for the heterogeneous TERT/ALT subgroup were unavailable at this timepoint. Median survival further highlighted differences among the groups: TERT-positive cases had a median survival of 3.5 years, while the ALT-positive subgroup showed a longer median survival of 5.6 years. In contrast, the TMM-negative sub-cohort and the small size of the heterogeneous TERT/ALT sub-cohort precluded the assessment of median survival. In both cases, fewer than 50% of patients experienced the event of interest within the observed timeframe, rendering the calculation of median survival time inapplicable. **(b)**

Event-free survival probabilities of the sub-cohorts. TERT-positive patients had a significantly shorter event-free survival than TMM-negative patients (67%). However, the 5-year event-free survival probability in the TERT-positive subgroup was 29% compared to 21% in the ALT-positive sub-cohort. The heterogeneous TERT/ ALT subgroup presented an event-free survival probability of 20% after 3-years of follow-up. Importantly, while the event-free survival probability of the TERT-positive and the TMM-negative subgroup remained the same at 10 years, this probability decreased to 18% in the ALT-positive subgroup, with a median event-free survival time of 1.8 years, and of 1.9 years for TERT-positive and heterogenous TERT/ALT sub-cohorts.
